# Development and cross-national investigation of a model explaining participation in WHO-recommended and placebo behaviours to prevent COVID-19 infection

**DOI:** 10.1038/s41598-022-17303-y

**Published:** 2022-10-21

**Authors:** Joanna Kłosowska, Elżbieta A. Bajcar, Helena Bieniek, Justyna Brączyk, Mohsen Joshanloo, Katia Mattarozzi, Arianna Bagnis, Moa Pontén, Maria Lalouni, Andrew L. Geers, Kelly S. Clemens, Joonha Park, Gahee Choi, Yun-Kyeung Choi, Wookyoung Jung, Eunjung Son, Hyae Young Yoon, Przemysław Bąbel

**Affiliations:** 1 Institute of Psychology, Jagiellonian University, ul. Ingardena 6, 30-060 Kraków, Poland; 2 Department of Psychology, Keimyung University, Daegu, Republic of Korea; 3 Department of Experimental, Diagnostic and Specialty Medicine, University of Bologna, Bologna, Italy; 4 Department of Clinical Neuroscience, Karolinska Institutet, Stockholm, Sweden; 5 Department of Psychology, University of Toledo, Toledo, USA; 6 NUCB Business School, Nagoya, Japan

**Keywords:** Psychology, Diseases, Risk factors

## Abstract

To protect themselves from COVID-19, people follow the recommendations of the authorities, but they also resort to placebos. To stop the virus, it is important to understand the factors underlying both types of preventive behaviour. This study examined whether our model (developed based on the Health Belief Model and the Transactional Model of Stress) can explain participation in WHO-recommended and placebo actions during the pandemic. Model was tested on a sample of 3346 participants from Italy, Japan, Poland, Korea, Sweden, and the US. It was broadly supported: objective risk and cues to action showed both direct and indirect (through perceived threat) associations with preventive behaviours. Moreover, locus of control, decision balance, health anxiety and preventive coping moderated these relationships. Numerous differences were also found between countries. We conclude that beliefs about control over health and perceived benefits of actions are critical to the development of interventions to improve adherence to recommendations.

## Introduction

In March 2020, the World Health Organization officially acknowledged the severe acute respiratory syndrome coronavirus (SARS-CoV-2) outbreak as a pandemic^1^ Governments responded to the spread of the disease by introducing regulations requiring citizens to take preventive measures that had been identified as critical to flattening the curve of the spread of COVID-19 (e.g., Ref.^2^), such as social distancing or facemasks. Even though effective COVID-19 vaccines have since been developed, preventive behaviours remain a fundamental strategy of stopping virus transmission^3^. Understanding which factors determine compliance with authorities’ guidance, especially at the beginning of the outbreak, is crucial for managing current and possible future pandemics.

There is growing evidence that to protect their own health people not only follow physicians’ recommendations but also employ placebo interventions, i.e., interventions that lead to a beneficial outcome after administration even if their active ingredients lack this potential^4^. Placebo interventions (e.g., homeopathic remedies) may influence some aspects of an individual’s health because the cues and rituals of the treatment trigger learned associations and expectations. These behaviours are quite common and are complementary rather than alternative to those recommended by physicians, at least when it comes to chronic conditions^5^. It seems that placebo actions may be a strategy for coping with the uncertainty^6^ that is characteristic of a pandemic, but they are likely not effective for virus protection. Rather, they may increase the spread of the virus if they outcompete WHO-recommended actions. Therefore, it is important to determine what psychological factors underlie use of placebos in this context.

A large number of studies conducted in behavioural sciences in the last 2 years focused on identifying sociodemographic, psychological and situational variables related to undertaking preventive behaviours and compliance with COVID-19 recommendations (e.g., Refs.^7–20^). Although our knowledge about the factors related to preventive behaviours during pandemic has increased considerably, the main limitation of existing studies is that they mostly focus on specific variables and are rarely based on health behaviour theories^21,22^. Therefore, our aim was to create and verify a model that is built on empirical knowledge and relevant theories that could help to understand the factors underlying individuals’ engagement with both recommended and placebo interventions.

**Figure 1:**
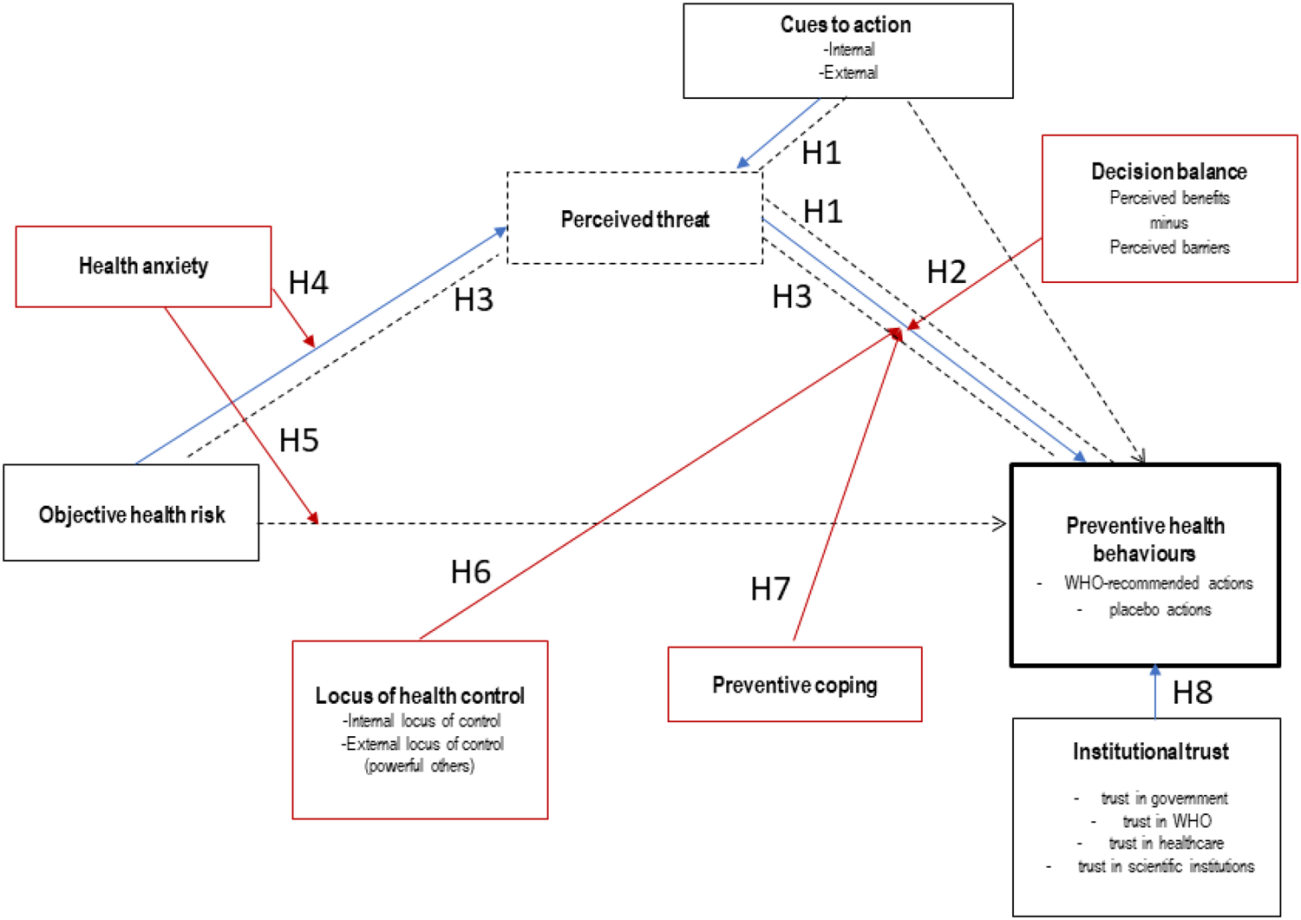
Hypothesized model. The model includes the following predictors: perceived threat of the COVID-19 disease (perceived susceptibility to infection and perceived severity of COVID-19), objective health risk (risk of developing serious symptoms of COVID-19), cues to action (internal and external cues that motivate an individual to act), decision balance (perceived barriers minus perceived benefits of action), individual characteristics (locus of health control, health anxiety and preventive coping style), institutional trust (including trust in government, WHO, healthcare and scientific institutions). Blue arrows represent direct effects. Moderators are represented by red rectangles; red arrows represent moderation effects. Dotted lines and dotted rectangles represent indirect effects and mediators, respectively.

In the current study we tested whether the model based on the Health Belief Model and the Transactional Model of Stress and Coping could be used to predict engagement in WHO-recommended actions and placebo actions during the COVID-19 pandemic. Figure 1 represents the factors included in the model and their interplay. Theoretical and empirical rationale for the hypothesized associations can be found in Supplementary Material A.

The following hypotheses were proposed:

H1: Positive relationship between cues to action and frequency of preventive behaviours is mediated by perceived threat.

H2: Decision balance moderates the relationship between perceived threat and frequency of preventive behaviours.

H3: Perceived threat mediates the relationship between objective risk of developing serious COVID-19 symptoms and the frequency of preventive actions.

H4: Health anxiety moderates the relationship between objective health risk and perceived threat.

H5: Health anxiety moderates the relationship between objective health risk and frequency of preventive actions.

H6: Health locus of control (powerful internal/external others) moderates the relationship between perceived threat and the frequency of preventive behaviours.

H7: Preventive coping style moderates the relationship between perceived threat and the frequency of engaging in preventive behaviours.

H8a: The frequency of WHO-recommended actions is positively associated with institutional trust.

H8b: The frequency of placebo actions is negatively associated with institutional trust.

The model was tested on an international sample consisting of participants who had not been diagnosed with COVID-19, recruited from 6 countries (Italy, Japan, Poland, Sweden, Republic of Korea, USA) that vary in cultural background as well as socio-political pandemic strategies. We wanted to determine (1) how the assumed model performs in the total sample as well as in subsamples recruited from different nations; (2) whether the model can be used to predict engagement in both WHO-recommended actions and placebo interventions.

## Results

### Preliminary analyses

The descriptive statistics and mean differences between nations are presented in Table 1 and the Supplementary Material C.

**Table 1:** Descriptive statistics (mean ± SD) and differences between countries.

	Italy	Japan	Korea	Poland	Sweden	USA	Differences	η^2^	Total sample
ITS healthcare	17.09 ± 3.17	15.83 ± 2.82	17.33 ± 2.88	14.70 ± 2.79	20.40 ± 3.03	17.07 ± 3.24	F(5,3340) = 287.17, p < 0.001	0.30	16.85 ± 3.55
ITS government	14.75 ± 4.17	13.10 ± 3.73	16.93 ± 4.68	12.04 ± 4.60	20.72 ± 3.87	11.50 ± 4.44	F(5,3340) = 389.63 p < 0.001	0.37	14.66 ± 5.50
ITS scientific institutions	20.26 ± 3.65	18.14 ± 2.78	18.83 ± 2.69	18.01 ± 2.72	23.24 ± 3.27	20.22 ± 3.83	F(5,3340) = 235.30 p < 0.001	0.26	19.68 ± 3.64
ITS WHO	19.36 ± 4.52	16.86 ± 3.39	15.20 ± 4.05	16.66 ± 4.19	22.14 ± 3.48	19.82 ± 4.48	F(5,3340) = 224.69 p < 0.001	0.25	18.19 ± 4.73
Institutional trust total	71.46 ± 12.83	63.92 ± 9.27	68.29 ± 9.41	61.40 ± 10.00	86.50 ± 10.63	68.61 ± 10.45	F(5,3340) = 454.78 p < 0.001	0.41	69.37 ± 13.51
Objective risk	0.69 ± 0.99	1.70 ± 0.55	2.31 ± 1.24	0.99 ± 1.11	1.69 ± 1.66	1.67 ± 1.54	F(5,3340) = 115.33 p < 0.001	0.15	1.44 ± 1.39
Subjective threat	48.33 ± 11.64	54.39 ± 13.22	57.64 ± 12.44	51.77 ± 15.19	39.36 ± 11.04	46.67 ± 14.45	F(5,3340) = 123.11 p < 0.001	0.16	49.55 ± 14.63
Cues to action	36.21 ± 4.90	30.27 ± 5.20	34.09 ± 5.66	32.51 ± 6.90	37.00 ± 4.57	36.78 ± 6.20	F(5,3340) = 87.51 p < 0.001	0.12	34.54 ± 6.29
Placebo actions	8.46 ± 6.21	9.44 ± 7.50	15.07 ± 6.31	13.85 ± 7.23	6.12 ± 4.43	12.21 ± 8.40	F(5,3340) = 150.86, p < 0.001	0.18	11.56 ± 7.50
WHO actions	39.21 ± 5.53	35.14 ± 6.28	36.68 ± 5.55	35.53 ± 8.69	35.67 ± 5.39	39.32 ± 6.21	F(5,3340) = 37.15, p < 0.001	0.05	36.76 ± 7.02
Internal LoC	24.44 ± 4.40	24.88 ± 4.66	26.43 ± 3.79	26.20 ± 4.80	22.93 ± 4.00	25.17 ± 5.35	F(5,3340) = 49.98, p < 0.001	0.07	25.22 ± 4.71
External LoC	18.78 ± 4.88	21.10 ± 4.27	23.42 ± 3.96	19.38 ± 6.14	17.48 ± 4.55	18.06 ± 6.15	F(5,3340) = 89.90, p < 0.001	0.12	19.55 ± 5.67
Preventive coping	28.69 ± 4.37	25.02 ± 5.43	28.30 ± 4.81	26.50 ± 5.33	29.25 ± 4.64	31.21 ± 6.33	F(5,3340) = 78.04, p < 0.001	0.11	28.19 ± 5.50
Health anxiety	34.04 ± 6.40	35.09 ± 6.98	34.78 ± 77.74	33.59 ± 8.39	29.97 ± 6.13	32.69 ± 10.12	F(5,3340) = 27.46, p < 0.001	0.04	33.16 ± 8.10
Decision balance	1.33 ± 0.94	0.86 ± 0.99	1.90 ± 1.00	1.13 ± 1.06	1.87 ± 0.82	1.75 ± 1.11	F(5,3340) = 87.93, p < 0.001	0.12	1.49 ± 1.07

Total sample: N = 3346; Italy: N = 405, Japan: N = 190, Republic of Korea: N = 551, Poland: N = 1092, Sweden: N = 587, USA: N = 521.

### Path analyses

**Figure 2:**
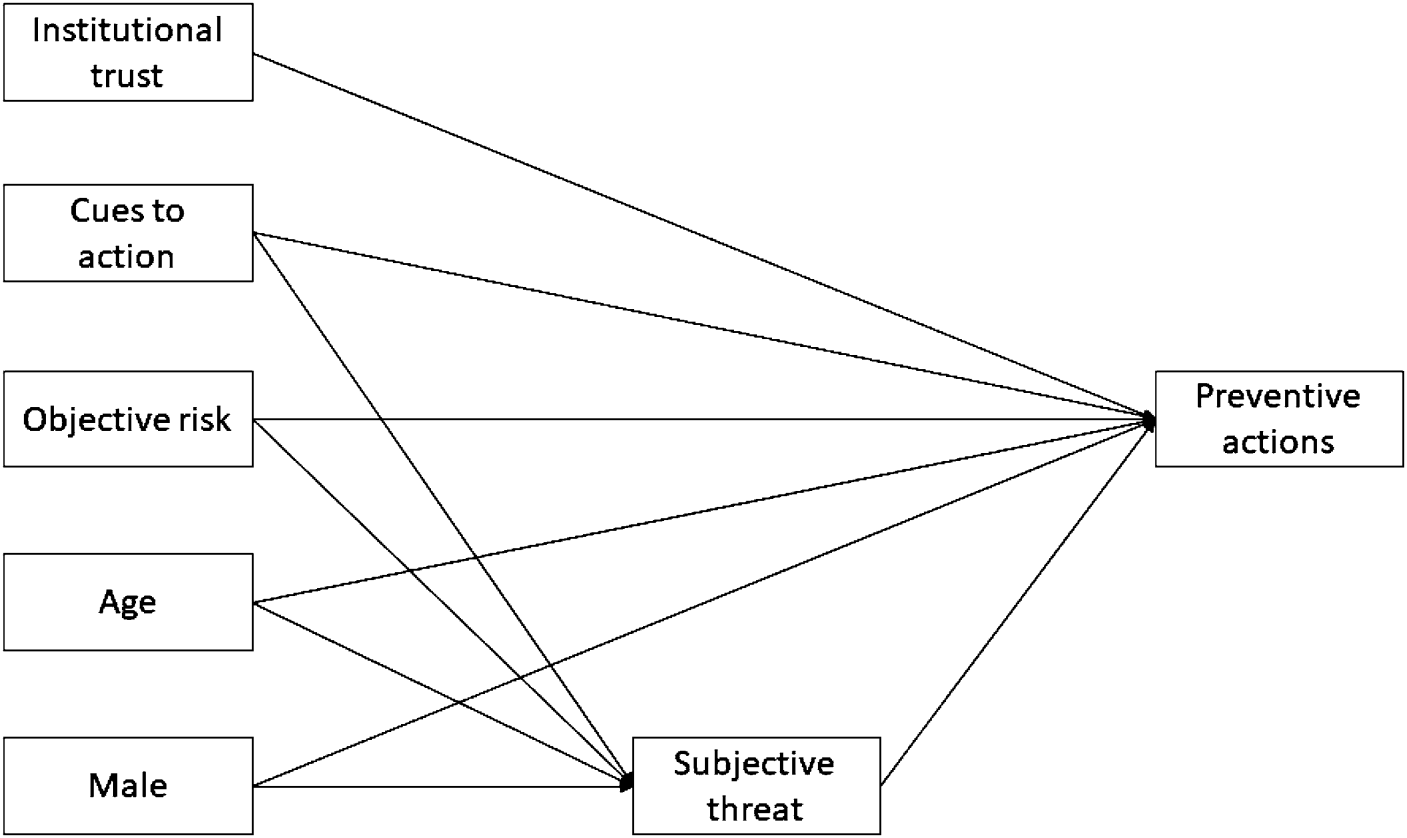
Baseline model. This model does not include moderators.

We started by fitting the baseline model of the study (as shown in Fig. 2) separately in each nation (excluding the moderators). Fit indices are reported in Table 2. As can be seen, the models provided an acceptable fit in Italy, Japan, Poland, and the USA. However, the fit was not acceptable in Korea and Sweden. The modification indices suggested that adding a covariance between subjective threat and institutional trust would substantially improve the fit in these two countries. Given the fact that previous studies showed a significant association between institutional trust and feeling of security, this covariance is theoretically justifiable^23^. The modified models provided an acceptable fit in Korea and Sweden. Next, we tested the two baseline models of the study in two multi-group analyses with all the countries, including the specified covariance for Korea and Sweden. As can be seen in Table 2, the two multi-group models provided an acceptable fit to the data. Together, these results suggest that the baseline models of the study are structurally comparable across the countries included in this study.

**Table 2:** Fit indices.

	*X*^2^	*df*	*p*	RMSEA (90% CI)	CFI	SRMR	Modifications/group-specific estimated parameters
**Single-country models**							
**WHO actions**							
Italy	1.778	1	0.182	0.044 (0.000–0.148)	0.994	0.017	–
Japan	0.070	1	0.792	0.000 (0.000–0.124)	1.000	0.005	–
Korea	14.799	4	0.005	0.069 (0.034–0.109)	0.952	0.041	Covariance between subjective threat and institutional trust
Poland	1.705	1	0.192	0.025 (0.000–0.089)	0.999	0.009	–
Sweden	5.948	4	0.203	0.029 (0.000–0.074)	0.991	0.021	Covariance between subjective threat and institutional trust
USA	0.085	1	0.770	0.000 (0.000–0.078)	1.000	0.003	–
**Placebo actions**							
Italy	1.778	1	0.182	0.044 (0.000–0.148)	0.994	0.017	–
Japan	0.070	1	0.792	0.000 (0.000–0.124)	1.000	0.005	–
Korea	14.800	4	0.005	0.069 (0.034–0.109)	0.930	0.040	Covariance between subjective threat and institutional trust
Poland	1.704	1	0.192	0.025 (0.000–0.089)	0.999	0.008	–
Sweden	5.948	4	0.203	0.029 (0.000–0.074)	0.988	0.021	Covariance between subjective threat and institutional trust
USA	0.085	1	0.770	0.000 (0.000–0.078)	1.000	0.003	–
**Multi-group model**							
WHO actions	139.824	28	0.000	0.085 (0.071–0.099)	0.933	0.052	Covariance between subjective threat and institutional trust for Korea and Sweden
Placebo actions	139.823	28	0.000	0.085 (0.071–0.099)	0.911	0.051	Covariance between subjective threat and institutional trust for Korea and Sweden

Total sample: N = 3346; Italy: N = 405, Japan: N = 190, Republic of Korea: N = 551, Poland: N = 1092, Sweden: N = 587, USA: N = 521.

It is worth noting that trust in institutions in the overall sample was positively associated with frequency of WHO actions (H8a) and negatively with placebo actions (H8b). Multi-group analyses showed that higher trust was significantly related to engagement in recommended behaviours only in Poland and Sweden, but not in other countries. A negative relationship between trust and placebo actions was detected in Italy, Sweden and USA. Interestingly, in Poland a positive association between institutional trust and placebo actions was observed. Regression coefficients are reported in Table 3.

**Table 3:** Results of the path analyses—unstandardized coefficients.

Parameter	Total sample	Italy	Japan	Korea	Poland	Sweden	USA
**WHO actions**							
Trust → WHO actions	0.030***	0.021	− 0.043	0.024	0.062**	0.053*	0.031
Cues → WHO actions	0.311***	0.188**	0.254*	0.334***	0.225***	0.195***	0.219***
Objective risk → WHO actions	0.064	− 0.525	0.202	− 0.247	− 0.068	0.416	0.493*
Subjective threat → WHO actions	0.142***	0.093***	0.123**	0.058*	0.230***	0.152***	0.108***
Age → WHO actions	0.006	0.095***	− 0.008	0.052*	0.035	0.011	− 0.016
Male → WHO actions	− 0.396	− 1.227	− 0.139	− 1.242*	− 1.684**	− 1.863**	2.744***
Cues → Subjective threat	0.667***	0.533***	0.654**	0.947***	1.222***	0.301**	1.102***
Objective risk → Subjective threat	2.381***	1.896**	− 3.565	0.276	1.227**	1.229**	0.946
Age → Subjective threat	− 0.235***	− 0.181***	− 0.224	0.015	0.157***	− 0.047	− 0.026
Male → Subjective threat	− 2.417***	− 9.346***	0.340	− 3.471**	− 4.782***	− 4.308***	3.383*
Trust → Subjective threat	− 44.723***	−	−	− 13.123**	−	− 43.358***	−
**Placebo actions**							
Trust → Placebo actions	− 0.127***	− 0.112***	0.050	0.011	0.067**	− 0.050**	− 0.158***
Cues → Placebo actions	0.133***	0.135*	0.083	0.323***	0.166***	0.113**	0.377***
Objective risk → Placebo actions	0.374**	0.339	0.615	− 0.593	0.699**	0.209	− 0.325
Subjective threat → Placebo actions	0.125***	0.034	0.123**	0.030	0.025	0.058**	0.141***
Age → Placebo actions	− 0.002	0.017	0.254*	0.101***	− 0.019	0.036	0.066*
Male → Placebo actions	− 0.127	− 2.044**	− 1.539	0.651	− 1.853***	− 0.335	− 1.548
Cues → Subjective threat	0.667***	0.533***	0.654**	0.947***	1.222***	0.301**	1.102***
Objective risk → Subjective threat	2.381***	1.896**	− 3.565	0.276	1.227**	1.229**	0.946
Age → Subjective threat	− 0.235***	− 0.181***	− 0.224	0.015	0.157***	− 0.047	− 0.026
Male → Subjective threat	− 2.417***	− 9.346***	0.340	− 3.471**	− 4.782***	− 4.308***	3.383*
Trust → Subjective threat	− 44.723***	–	–	− 13.122**	−	− 43.358***	–

*p < 0.05. **p < 0.01. ***p < 0.001; Total sample: N = 3346; Italy: N = 405, Japan: N = 190, Republic of Korea: N = 551, Poland : N = 1092, Sweden: N = 587, USA: N = 521; WHO actions: R^2^ = 0.23 for the total sample, R^2^ = 0.14 for Italy, R^2^ = 0.14 for Japan, R^2^ = 0.21 for Korea, R^2^ = 0.32 for Poland, R^2^ = 0.17 for Sweden, R^2^ = 0.25 for the USA; Placebo actions: R^2^ = 0.13 for the total sample, R^2^ = 0.11 for Italy, R^2^ = 0.08 for Japan, R^2^ = 0.13 for Korea, R^2^ = 0.08 for Poland, R^2^ = 0.11 for Sweden, R^2^ = 0.21 for the USA.

There were differences in the sizes of the regression coefficients across the countries. Accordingly, we decided to run our mediation and moderated mediation analyses not only in the total sample but also separately in each country. The results of the analyses conducted in different nations are described in Supplementary Material.

### Mediation analyses

In line with H3, in total sample, subjective threat mediated the relationship between objective risk of developing serious symptoms of COVID-19 and the frequency of WHO-recommended actions (indirect effect: b = 0.44, SE = 0.05, 95% CI 0.35–0.54) as well as placebo actions (b = 0.40, SE = 0.05, 95% CI 0.31–0.49). The total effects of objective risk on both types of preventive actions were positive and significant. After controlling for subjective threat, the relationship between objective risk and WHO-recommended actions became non-significant. The effect of objective risk on placebo actions was still significant; however, it became weaker, thus suggesting partial mediation. Furthermore, as hypothesized (H1), the indirect effects of cues to action through subjective threat on both types of preventive behaviours were also significant (WHO actions: b = 0.09, SE = 0.01, 95% CI 0.08–0.11; placebo actions: b = 0.11, SE = 0.01, 95% CI 0.09–0.13). Results of hypotheses testing are summarized in Table 4. Results of mediation analyses conducted separately in each of the six studied nations are presented in Supplementary Materials D and E.

**Table 4:** Results of hypotheses testing.

Hypothesis	Total sample	Italy	Japan	Korea	Poland	Sweden	USA
**WHO actions**							
H1	✓	✓	✓	✓	✓	✓	✓
H2	✓	✓	✓	✓	✓	✓	
H3	✓	✓			✓	✓	
H4							✓
H5							
H6	✓				✓		
H7							
H8a	✓				✓	✓	
**Placebo actions**							
H1	✓		✓			✓	✓
H2					✓		✓
H3	✓	✓			✓	✓	
H4							✓
H5	✓		✓				✓
H6	✓			✓	✓	✓	✓
H7	✓				✓		
H8b	✓	✓			?	✓	✓

✓—confirmed, ?—correlation was significant but positive (not negative, as predicted).

### Moderated mediation analyses

**Figure 3:**
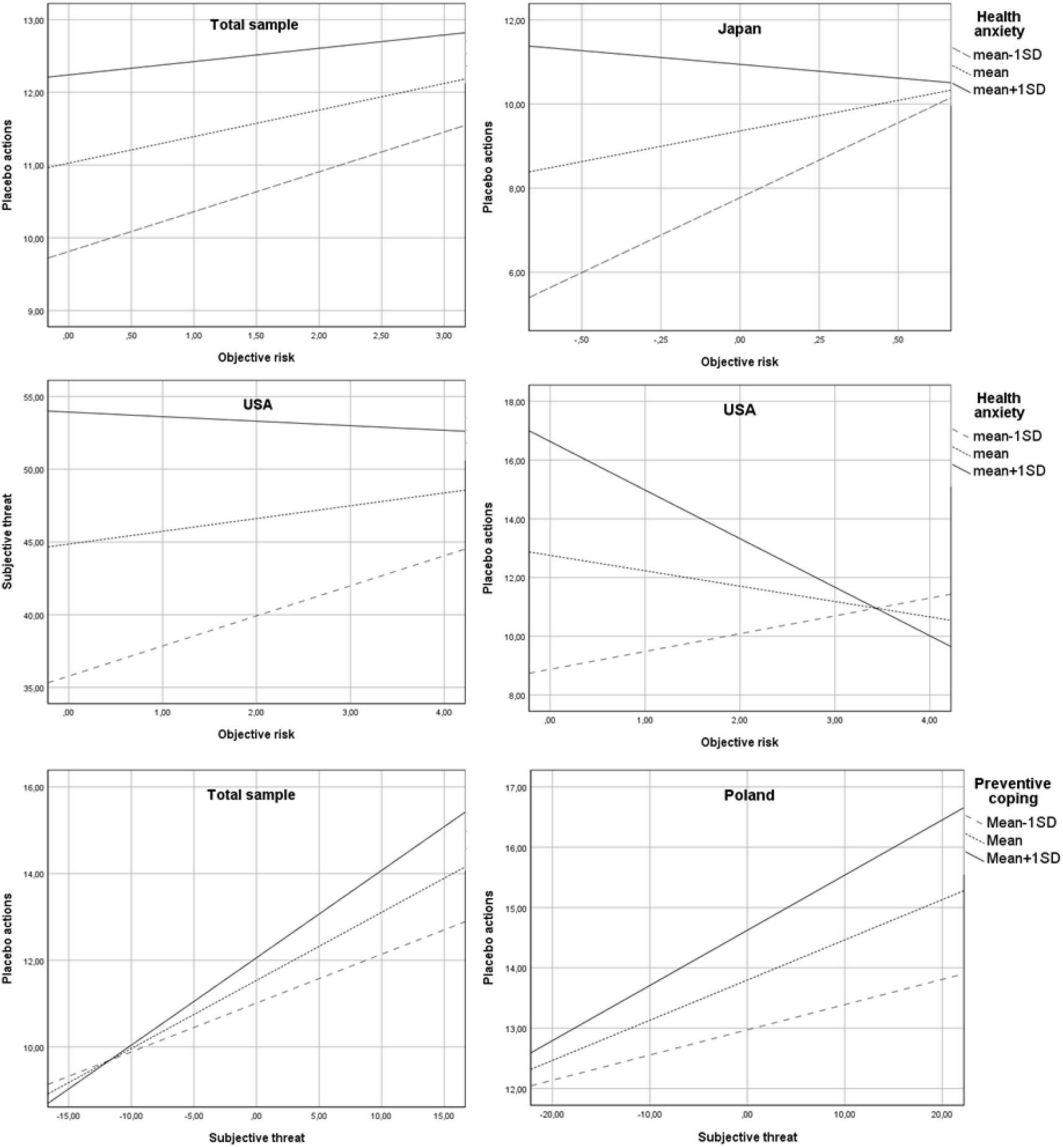
Simple slope analyses (hypotheses 4, 5 and 7). The interaction effect of objective risk and illness anxiety on subjective threat (H4) was found only in the USA. The relationship between objective risk and subjective threat was positive and significant only among Americans with low (mean − 1SD) levels of health anxiety. The moderation effect of illness anxiety on the relationship between objective risk and preventive actions (H5) was confirmed in the USA and Japan and only in the case of placebo actions: in Japan, the positive relationship between objective risk and frequency of placebo actions was stronger for individuals characterized by low (mean − 1SD) levels of health anxiety than those characterized by higher levels, the opposite pattern was found in the USA. The interaction effect of subjective threat and preventive coping style on preventive behaviours (H7) was confirmed only in Poland, and only for placebo actions. The positive relationship between subjective threat and frequency of engaging in placebo actions was stronger among individuals characterized by high (mean + 1SD) levels of preventive coping style than in individuals characterized by lower levels; Total sample: N = 3346; Japan: N = 190, Poland: N = 1092, USA: N = 521.

In the total sample, the hypothesized interaction effect of objective risk and health anxiety on subjective threat (H4) was not confirmed for any type of preventive behaviours. Similarly, health anxiety did not moderate the relationship between objective risk and frequency of WHO-recommended actions (H5). However, the interaction effect of objective risk and heath anxiety on frequency of placebo actions was significant (b = − 0.02, SE = 0.01, 95% CI − 0.04 to − 0.00, p < 0.05). Specifically, the positive relationship between objective risk and frequency of placebo actions was stronger at low (Mean − 1SD) levels of health anxiety (see Fig. 3) than at higher levels of health anxiety.

**Figure 4:**
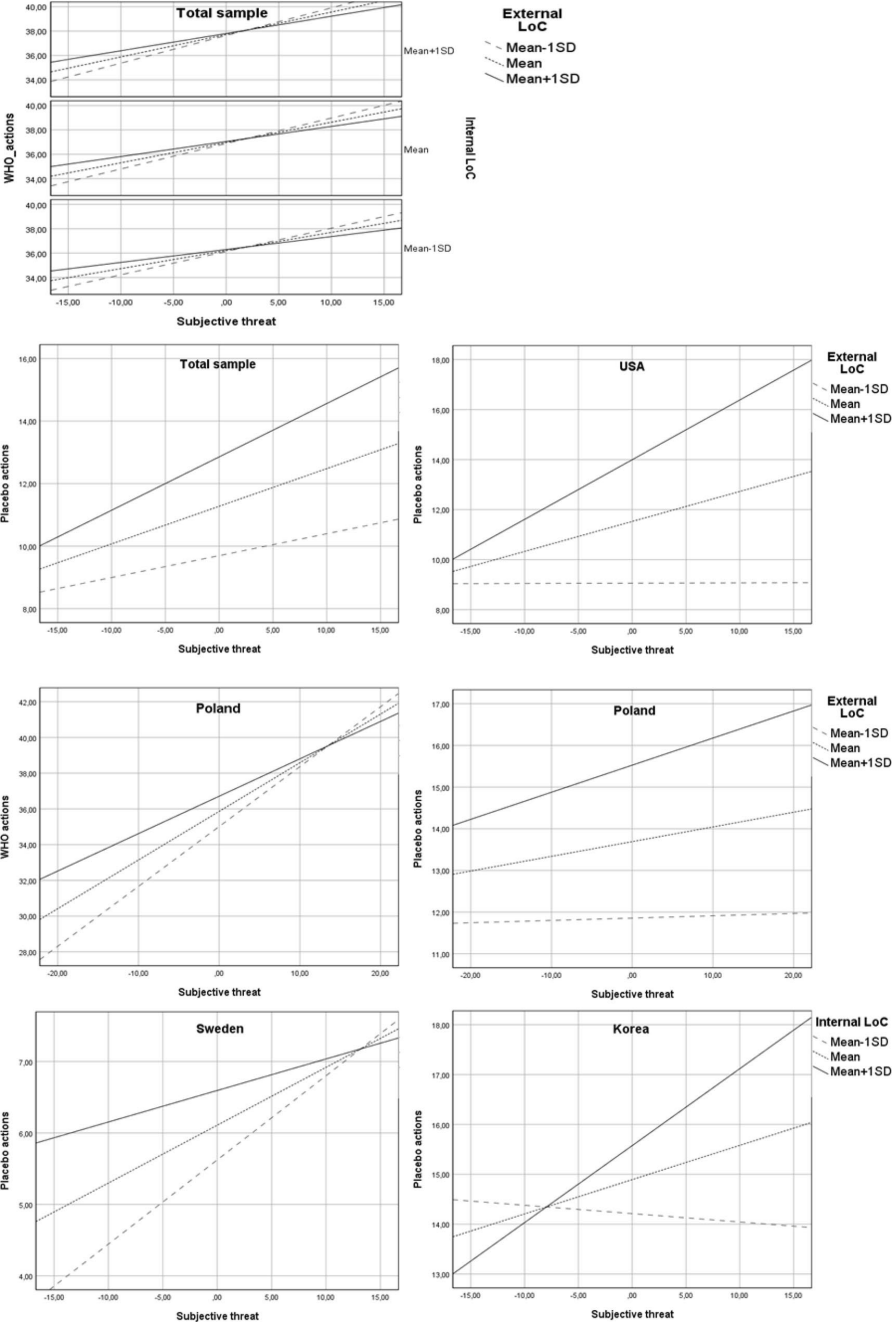
Simple slope analyses (hypothesis 6). The interaction effect of subjective threat and locus of health control (H6) was confirmed in Poland, Republic of Korea, Sweden and USA. In Poland, the relationship between subjective threat and both types of preventive actions was moderated by the external locus of health control (external LoC). The effect of subjective threat on the frequency of WHO-recommended actions was reduced in the case of individuals with high (mean + 1SD) levels of external LoC. Additionally, the mediation effect of objective risk through subjective threat on WHO actions was significant only in participants who reported low (mean − 1SD) levels of external LoC but not in those who reported medium (mean) or high levels. The positive effect of subjective threat on placebo actions was reduced among participants with low levels of external LoC. In the USA, the interaction effect of subjective threat and external LoC on placebo actions was significant: the relationship between feeling threatened and the frequency of applying placebo interventions was positive and was stronger in individuals characterized by high levels of external LoC than in individuals characterized by medium or low levels. In Republic of Korea and Sweden there was a significant interaction effect of subjective threat and internal locus of health control (internal LoC) on frequency of placebo actions. In Sweden, a stronger positive effect of subjective threat on frequency of placebo actions was observed among individuals with low levels of internal LoC than in those with medium and high levels. In the Republic of Korea, the positive effect of perceived threat was stronger among people with higher levels of internal LoC than among people with lower levels; Total sample: N = 3346; Republic of Korea: N = 551, Poland: N = 1092, Sweden: N = 587, USA: N = 521.

Both external and internal locus of health control (LoC) moderated the relationship between subjective threat and frequency of WHO-recommended actions (H6) (internal LoC: b = 0.004, SE = 0.00, 95% CI 0.00–0.01, p < 0.05; external LoC: b = − 0.01, SE = 0.00, 95% CI − 0.01 to − 0.00, p < 0.001). The effect of subjective threat on the frequency of WHO actions was stronger in individuals with a high level of internal LoC and a low level of external LoC than in individuals with lower levels of internal LoC and higher levels of external LoC, respectively. Moreover, the indirect effect of objective risk through subjective threat was stronger at lower levels of external LoC (index of moderated mediation = − 0.02, SE = 0.01, 95% CI − 0.03 to − 0.01) than at higher levels. Additionally, there was a significant interaction effect of external LoC and subjective threat on frequency of placebo actions (b = 0.01, SE = 0.00, 95% CI 0.005–0.01, p < 0.001). The positive relationship between threat and placebo actions was stronger for participants with high levels of external LoC (Fig. 4) than participants with medium and low levels. The indirect effect of objective risk through subjective threat was stronger at higher (mean + 1SD) levels of the moderator variable than at lower levels (index of moderated mediation: 0.02, SE = 0.00, 95% CI 0.01–0.03).

The interaction effect of subjective threat and preventive coping (H7) was confirmed only for placebo actions (b = 0.01, SE = 0.00, 95% CI 0.005–0.01, p < 0.001). The effect of subjective threat on placebo actions was reduced in individuals showing low levels of this coping style (Fig. 3). Furthermore, the indirect effect of objective risk through subjective threat was stronger at higher (mean + 1SD) levels of preventive coping (index of moderated mediation: b = 0.02, SE = 0.00, 95% CI 0.01–0.03) and weaker at low levels of preventive coping.

**Figure 5:**
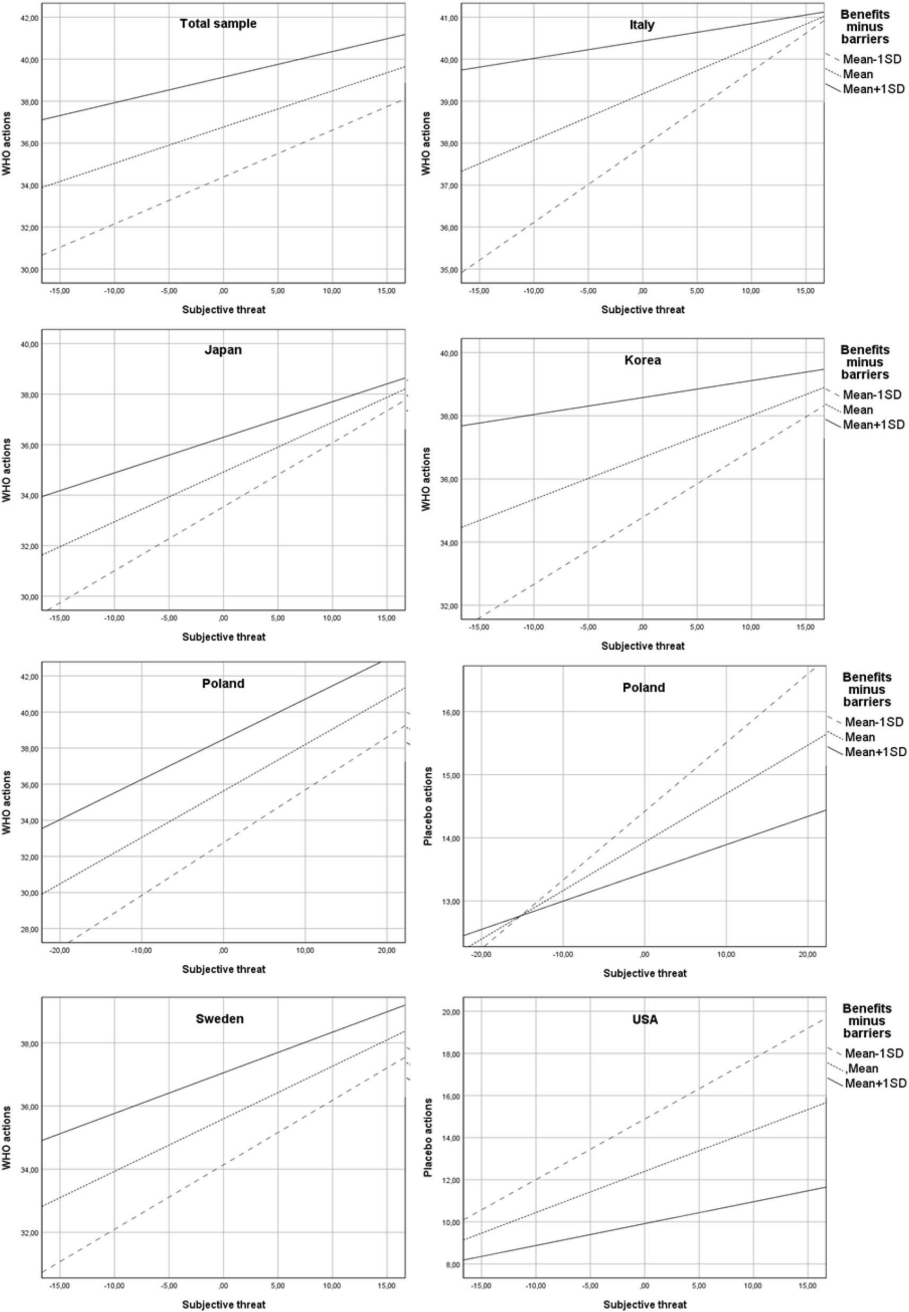
Simple slope analysis (hypothesis 2). Note: The interaction effect of subjective threat and decision balance (H2) was present in all of the analysed nations. However, in most cases this effect was confirmed only for one type of preventive actions and not the other. The only exception was Poland, where a significant interaction effect was found for both WHO-recommended actions and placebo actions. A reduced positive effect of perceived threat on frequency of both types of behaviours was observed among individuals who perceived more benefits than barriers associated with preventive actions. In the case of WHO-recommended actions, a similar effect was observed in Japan, Italy, Sweden and the Republic of Korea. In the USA, this effect was found only in the case of placebo actions. Total sample: N = 3346; Italy: N = 405, Japan: N = 190, Republic of Korea: N = 551, Poland: N = 1092, Sweden: N = 587, USA: N = 521.

Finally, there was a significant interaction effect of subjective threat and decisional balance (H2) on WHO-recommended actions (b = − 0.05, SE = 0.01, 95% CI − 0.06 to − 0.04, p < 0.001). Namely, the effect of subjective threat on frequency of undertaken actions was weaker in individuals who perceived more benefits than barriers than in those who saw more barriers than benefits to preventive actions (Fig. 5). The indirect effect of objective risk through perceived threat was the strongest when a person declared that he/she saw more barriers than benefits associated with following WHO recommendations (index of moderated mediation: − 0.12, SE = 0.02, 95% CI − 0.16 to − 0.08). The results of the analyses are summarized in Supplementary Material F. Moreover, the results of moderated mediation analyses conducted separately in each nation are described in the Supplementary Material D.

## Discussion

As hypothesized, in the total sample, subjective threat was found to fully mediate the link between objective risk and frequency of WHO-recommended actions. Moreover, the relationship between objective risk and frequency of placebo actions was partially mediated by subjective threat. The latter also served as a partial mediator in the relationship between cues to actions and both types of preventive behaviours. Health anxiety moderated the relationship between objective risk and frequency of placebo actions, but this effect was not detected for WHO-recommended actions. Both external and internal LoC were found to be moderators of the link between subjective threat and WHO-recommended actions, whereas the relationship between subjective threat and placebo actions was moderated only by external LoC. Also, an interaction effect of subjective threat and preventing coping was detected, but only in placebo actions. On the other hand, decision balance moderated the subjective threat–preventive behaviours relationship only in the case of WHO-recommended actions. Institutional trust was positively related to the recommended behaviours and negatively to placebo interventions. However, contrary to our expectations, the hypothesized interaction effect of objective risk and health anxiety on the level of subjective threat was not confirmed for any type of preventive behaviours.

There was a positive relationship between subjective risk and engagement in authorities’ recommendations. Similar results were obtained in previous studies conducted during the COVID-19 pandemic^10,13,18^. Moreover, in line with our hypotheses, subjective risk served as a mediator in the relationship between objective risk of developing serious symptoms of COVID and frequency of taking recommended precautions. Perceived risk also mediated the link between cues to actions and recommended behaviours. These results support the thesis, which was formulated on the basis of both Health Belief Model^24–26^, and Transactional Model of Stress^27,28^ that situational factors motivate to act through shaping a person’s beliefs; in this case, beliefs about vulnerability to coronavirus infection and the seriousness of COVID-19.

The study also confirmed recent findings^29–34^ and showed that engagement in recommended actions is positively associated with institutional trust. Moreover, as predicted, decision balance moderated the relationship between subjective risk and frequency of following recommendations: people who perceived more benefits than barriers to actions engaged in recommended behaviours more frequently than those who saw more barriers. At the same time, the relationship between subjective risk and frequency of following recommendations was stronger when people perceived more barriers than benefits of actions. This result is in the line with the hypothesis formulated by Champion and Skinner^26^, who speculated that when people see few barriers and many benefits, they may engage in preventive behaviours even if the threat is not very high. The motivating role of perceived threat becomes visible when the number of perceived barriers increases and behaviour is seen as costly and non-beneficial.

Also, the LoC served as a moderator in the relationship between subjective risk and frequency of following recommendations. In particular, the strongest link between subjective risk and engagement in recommended behaviours was observed among people with high levels of internal LoC and low levels of external LoC. This result is in line with many previous studies showing that increased internal LoC and decreased external LoC are associated with intensified engagement in pro-health and preventive behaviours^35,36^ as well as higher compliance with protective COVID-19 guidelines^37^.

Contrary to our predictions, the moderating role of health anxiety in the relationship between objective threat and frequency of engagement in recommended actions was not supported. Similarly, the interaction effect of subjective risk and preventive coping style on recommended behaviours was not detected in the total sample. It is possible that the role of these individual characteristics in shaping health behaviours becomes visible when actions are mainly the effect of personal preferences, not the effect of external pressure. Some of the behaviours recommended during the pandemic were required and enforced by law in some countries participating in the study. As a result, individuals whose preferences would not lead them to engage in such behaviours would still willingly do so due to fear of the possible penalty for non-compliance.

This explanation is partially supported by the fact that the hypothesized interaction effects involving health anxiety and preventive coping were detected in the case of placebo actions, which are mostly chosen by individuals and are usually not required. Specifically, it was found that the relationship between perceived threat and placebo actions is stronger for people who prefer to build resistance “just in case” something bad happens than for those with a low level of future-oriented coping style. Additionally, the results suggest that people with high levels of health anxiety frequently engage in placebo actions, regardless of the level of objective health threat; this is in line with the notion that these individuals are extra-sensitive to signals of danger and sometimes interpret neutral bodily signs as threatening symptoms^38,39^. On the other hand, for people with lower levels of health anxiety, the greater the objective danger of a severe course of COVID-19, the more preventive placebo interventions they use.

As hypothesized, the relationship between trust in institutions and the frequency of placebo actions in the total sample was negative, showing that mistrust in organisations that provide information about efficient types of protection against the virus may be one of the reasons that people engage in placebo actions. As we know, conspiracies theories emerged very soon after the pandemic started, many of which focused on different ways of warding off the virus and alternative remedies for the symptoms of COVID-19^40,41^. The recent findings of Banai et al.^42^, Chan et al.^43^ and Pummerer et al.^44^ show that believing in COVID-19 conspiracy theories may decrease institutional trust and adoption of recommended preventive measures. The results of our study tentatively suggest that conspiracies theories may also lead to an increase in the usage of placebo interventions due to the spread of public distrust. This link should be further explored in future studies.

The strongest relationship between subjective risk and the frequency of using placebo interventions was found among individuals with high levels of external LoC. Internal LoC did not moderate this relationship. The pandemic seems to be especially threatening for people who do not feel in control of their own life. For example, studies have shown that external LoC aggravates the relation between COVID-19-related stress and symptoms of depression and anxiety^45,46^. Interestingly, external LoC has also previously been found to correlate positively with the tendency to believe in conspiracy theories^47,48^. For people who do not feel in control of their health, conspiracy beliefs may be a way of alleviating anxiety by reassuring them that the world in not completely random and unpredictable^49^. It is therefore possible that people with high levels of external LoC are more prone to conspiracy theories, therefore they are more likely to use placebo interventions when feeling at risk of disease.

In contrast to the WHO-recommended actions, the moderating role of decision balance in the relationship between subjective threat and frequency of engaging in placebo actions was not observed. This result is somewhat surprising; however, it is possible that when asked about the perceived benefits of preventive behaviours and the barriers associated with them, participants referred mainly to recommended actions and not to placebo interventions. The pros and cons of following recommended precautions are widely discussed in the media as well as in everyday life, therefore this kind of information is easy to access when formulating opinions, but the same cannot be said about most placebo interventions. In other words, despite our efforts to create neutral instructions that would not suggest any type of action, due to participants’ assumptions the “decision balance” variable in our study might have in fact reflected people’s beliefs about the pros and cons of following authorities’ recommendations and therefore might play a lesser role in determining engagement in placebo actions.

Finally, the interaction effect of objective risk and health anxiety on the level of subjective risk was not confirmed in the total sample for any type of preventive behaviour. The only country where this effect was detected was the United States, which was experiencing the second wave of the pandemic at the time of the study. During this time period, the epidemiological situation in the US was serious, and American citizens were bombarded by media with many distressing pictures showing coronavirus victims. It is therefore possible that participants high on health anxiety misinterpret somatic signals as possible symptoms of COVID-19 and feel endangered only when COVID-19 disease is perceived by them as highly prevalent condition and therefore probable cause of their symptoms. When it is not the case, they may instead focus on other possible health risks and diagnoses which they perceive as threatening. That would explain why the moderating role of health anxiety in objective risk-subjective threat relationship was not detected in countries, which at the time of data collection did not have many active cases of COVID-19. The further discussion of the findings obtained in different studied nations can be found in Supplementary Material I.

### Strengths, limitations and future directions

The current study has a number of advantages that needs to be acknowledged. First, we developed and tested a comprehensive and theoretically grounded model that explains engagement in preventive behaviours during the pandemic. What especially distinguishes this model from other health behaviour models is the fact that it not only incorporates psychological constructs but also considers biological factors, namely the objective risk of developing serious symptoms of COVID-19. Additionally, we did not settle for analysing simple associations between constructs but also endeavoured to specify how these variables combine and interact with each other to produce behaviour. Our study is also the first to investigate the predictors of engagement in placebo interventions during the pandemic. Furthermore, it is worth noting that the research was conducted in a large international sample consisting of participants from six culturally diverse countries that deployed various policies to combat the pandemic. This enabled us to test our model in different socio-cultural and epidemiological contexts.

Some limitations need to be acknowledged when interpreting the results; these should be addressed in future research. The study was cross-sectional, which limits the possibility of casual inference. Longitudinal and experimental studies should be performed to further validate the proposed model. However, it is worth mentioning that the model was formulated on the basis of well-established theories, which strictly guided the assumed directions of the hypothesized associations (see Supplementary Material A for further details). Furthermore, engagement in preventive behaviours was assessed retrospectively, which introduced the possibility of recall bias. In the future, more precise methods should be used to collect information about behaviour, such as a diary method with daily entries. We used internet advertisements and social media to recruit participants from different countries; as a result, older adults, who are not always active internet users, were underrepresented. The subsamples’ demographic composition differed, including differences in age and sex. The subsamples also differed in size: for example, the sample from Japan was much smaller than the rest (and therefore underpowered). In future, more balanced samples are recommended. Moreover, due to problems with recruiting participants in the United States, data collection took more time in this country. As a result, the study was mostly conducted during the second wave of the pandemic (in other countries, the study was conducted after the first wave, when governments loosened restrictions). Although it would be preferable to collect all data at the same time, we were able to verify how our model would perform in different epidemiological contexts. We did not investigate the determinants of vaccine uptake, which is undoubtedly a very important measure to prevent the development of COVID-19 symptoms^50,51^ because the approved SARS-CoV vaccines had not yet been available at the time of the data collection. Future research should verify if the proposed model also explains vaccination behaviour. Finally, it should be pointed out, that the results of our study probably cannot be generalised to the developing countries with a less-integrated health system, where local organizations and response at grassroot levels play a crucial role in citizens’ response to the epidemic^52^.

### Implications for practice

The findings of this study may be used to guide interventions aimed at increasing compliance with authorities’ recommendations during viral outbreaks. The results suggest that higher objective risk of developing serious symptoms does not necessarily translate into undertaking more preventive behaviours if a person does not perceive himself/herself as at risk. They also show that it may not be enough to increase the level of perceived threat associated with coronavirus (for example, by showing the negative consequences of COVID-19 in the media) to make people follow recommendations. In order to act, people also need to feel like they have control over their health and perceive more benefits than barriers associated with actions. Therefore, campaigns should also focus on these positive factors highlighting the fact that individuals are responsible for their own health and may effectively protect themselves against coronavirus infection, as well as showing what may be gained (by the individual as well as others) if the precautions are implemented. Some of the practical and psychological barriers that discourage people from following recommendations can also be addressed by making preventive measures more affordable, easily available and socially acceptable. The last goal may be achieved for example by presenting individuals with accurate information about the acceptance and prevalence of different preventive actions^53^. Increasing public trust is also important because, as our study shows, individuals need to trust institutions if they are to follow their lead. Otherwise, they may look for other not scientifically proven methods of protection against infection. It has been previously pointed out^54^ that proper delivery of public health crisis communications is crucial in enlisting public trust and cooperation during epidemics. Transparency, consistency, credibility, sensitiveness to the concerns and values of diverse public, as well as acknowledgment of uncertainties are some of the characteristics of effective government crisis communication that may have positive impact on institutional trust^54^.

## Conclusions

Drawing upon theory and earlier empirical findings, we proposed a model that explains engagement in protective actions during the pandemic and tested it in a diverse sample that included participants from six different countries. Overall, the model performed quite well in the total sample; however, many differences between nations were also detected (see Supplementary Materials), suggesting that situational and socio-cultural factors cannot be ignored when explaining health behaviours during the COVID-19 pandemic. Especially, the predictors of preventive actions in nations representing collective cultures need to be further investigated as both current and previous studies (e.g. Refs.^55,56^) suggest they can be different from those identified in countries representing individualistic cultures. It might not be a coincidence that the theories that inspired our model, i.e., HBM and TMS, were both created by American psychologists, and USA was one of the nations with the highest ratio of confirmed hypotheses in our study. As was previously pointed out by different authors (e.g., Ref.^57^), theories that work well in a particular socio-cultural context cannot necessarily be generalized to other contexts. Further studies should focus on determinants of preventive behaviours that are more specific to other cultures that are not incorporated into the models such as HBM or TMS. Previous research suggests that in the case of collective cultures these factors may for example include the belief that others find it important to engage in preventive actions^58^, anticipated stigma, or fear of offending and distressing others^59^. Also, it is worth mentioning that in addition to collectivism-individualism, other dimensions associated with culture such as uncertainty avoidance^60^, tightness-looseness^61^ or honour culture^62^ can be important for understanding adherence to preventive behaviours during epidemics^55,63^.

## Methods

### Participants

The initial sample consisted of 3681 participants (54.1% women, 45.6% men, 0.2% other) who completed the surveys. 265 participants were excluded from the analyses because they incorrectly answered the control questions (e.g., “In this question, please select 3”). A further 79 participants were excluded because they declared they had COVID-19 at the time of the study (26 participants), or they had already been diagnosed with COVID-19. The final sample included 3346 participants from six countries: Italy (405, 12.1%), Japan (190, 5.7%), Republic of Korea (551, 16.5%), Poland (1092, 32.6%), Sweden (587, 17.3%), the United States (521, 15.6%). Of these, 54.2% were women. The declared age of participants ranged from 18 to 89 years old (M = 38.10, SD = 15.76). The study was conducted from June 2020 to December 2020. Participants were recruited in each country via internet advertisements and social media. The sociodemographic characteristics of the sample are presented in Supplementary Material B.

The study consisted of completing an online survey comprised of a series of questionnaires, implemented by Qualtrics software. At the beginning of the survey, the participants were informed about the purpose of the study, the time needed to complete the questionnaires, and the types of questions they would encounter. Moreover, they were assured of full data confidentiality and anonymity, their use for scientific purposes only, and the possibility to stop participation in the study at any time by simply closing the browser window. Then, the participants gave informed consent to participate in the study by ticking the relevant box. The study protocol was reviewed and approved by the Ethical Committee of the lead researcher’s faculty (Research Ethics Committee at the Institute of Psychology, Jagiellonian University, number: KE/01/062020) as well as committees of Partner Universities (University of Bologna Ethical Committee, number: 0017109; The University of Toledo Social, Behavioural and Educational, IRB number: 300706). The study and all methods have been performed in accordance with the Declaration of Helsinki.

### Materials and procedures

The following variables were measured: (1) demographic data (sex, age, ethnicity, marital status, education, employment status, income, country); (2) objective risk of developing serious COVID-19 symptoms; (3) subjective threat posed by COVID; (4) internal and external cues that motivate an individual to take action; (5) institutional trust; (6) WHO-recommended actions and placebo interventions undertaken to prevent viral infection; (7) perceived benefits and barriers of preventive actions; (8) anxiety, locus of control, coping; and (9) a 7-item lie scale to make sure participants were careful and attentive when completing the questionnaires.

#### The objective health risk

The objective health risk index was calculated based on the Objective Risk Stratification Tool, developed by Strain and collaborators^64^. Demographic characteristics, such as age, sex, and ethnicity were included, along with obesity, chronic diseases and immunosuppressant therapy.

The variables numbered from 3 to 7 were measured by questionnaires developed for the purposes of the study:

#### Subjective threat posed by the illness

This questionnaire contains 16 items that assess how much a person is afraid that being infected with SARS-COV-2 would seriously impact their life (health, work situation, etc.). In the current sample, Cronbach’s alpha for the total scale was 0.94.

#### Internal and external cues that motivate an individual to take action

This questionnaire contains 11 items that assess a person’s exposure to stimuli or situations that encourage the use of SARS-COV-2 preventive measures. Cronbach’s alpha calculated for the current sample was 0.69.

#### Institutional trust

This questionnaire contains 22 items that assess the degree to which a person trusts institutions involved in SARS-COV-2 prevention. It contains four subscales: Trust in healthcare institutions, Trust in government, Trust in scientific institutions, and Trust in the World Health Organization. In the studied sample, Cronbach’s alpha for the total scale was 0.90.

#### WHO-recommended actions and placebo interventions undertaken to prevent viral infection

This questionnaire contains a list of 33 actions that may be used for anti-viral purposes: twelve WHO-recommended actions (e.g., wearing masks in public places, washing hands with soap and water), ten placebo actions (taking homeopathic remedies, using essential oils) and eight other, health‑related behaviours (e.g., exercising, avoiding alcohol). The scale measures to what extent an individual has been implementing these types of preventive actions. In this study we focused on frequency of engaging in placebo and WHO recommended actions; other health-related behaviours were not analysed. Cronbach’s alpha for the subscale measuring WHO recommended action was 0.77 and for the subscale measuring placebo actions 0.81.

#### Perceived benefits and barriers of preventive actions

This questionnaire contains 15 items divided into two subscales (perceived benefits from behaviours and perceived barriers to behaviours) that measure to what extent a person sees the use of preventive behaviours in general as helpful and necessary (e.g. “Engaging in preventive actions keeps other people around me from catching COVID-19”, “Engaging in preventive actions allows me to do my job despite current circumstances”), or burdensome (e.g. “Engaging in preventive actions is very time-consuming”, “Engaging in preventive actions makes me a laughing stock”). In the studied sample, Cronbach’s alpha for the perceived benefits subscale was 0.78 and for perceived barriers 0.86.

Items in each of these questionnaires were measured on a 5-point Likert scale. The exact wording of the items from all question sets can be found in Supplementary Material J.

#### *Short Health Anxiety Inventory* (SHAI)^65^

The SHAI contains 18 items that assess concerns about health, awareness of bodily sensations or changes, and feared consequences of having an illness. Each item is scored on a scale from 0 to 3 (i.e., a = 0, b = 1, c = 2, d = 3). In the current sample, Cronbach’s alpha for the total scale was 0.89.

#### Multidimensional Health Locus of Control, Form A (MHLC-A)^66^

The MHLC-A is an 18-item scale that measures the degree to which an individual believes that his or her health behaviour is controlled by external or internal factors. It contains three 6-item subscales: Internal Health Locus of Control (Cronbach’s alpha in the studied sample = 0.70), Powerful Others Health Locus of Control (Cronbach’s alpha = 0.71), and Chance Health Locus of Control (Cronbach’s alpha = 0.72). Each of these subscales contains six items with a 6-point Likert response scale.

#### The Preventive Coping Subscale of The Proactive Coping Inventory (PCS)^67^

Preventive coping deals with anticipation of potential stressors and the initiation of preparation before these stressors develop fully. The PCS-subscale contains 10 items which are rated on a 4-point Likert scale. The Cronbach’s alpha = 0.87 in the current sample.

As our study was part of a larger project, the respondents also completed the Perceived Stress Scale^68^ the Perceived Vulnerability to Disease Scale^69^, and the Cyberchondria Severity Scale^70^, none of which were included in the presented model.

The survey was created in English and then translated into and adjusted for (e.g., to country regions) each language by native speakers from our research team. The relevant language version and validation of the psychological questionnaires were used in each country. If a translation and validation of certain psychological questionnaires were not available, the questions were translated from the English version by native speakers.

### Statistical analyses

Mplus 8.4 was used to run multigroup path analyses. All path models were estimated with robust maximum likelihood (MLR) using all available data. The following values were considered as indicative of an acceptable model fit (e.g., Ref.^71^): minimum cut-off of 0.90 for the comparative fit index (CFI); maximum cut-off of 0.07 for the root mean square error of approximation (RMSEA); maximum cut-off of 0.08 for the standard root mean square residual (SRMR).

To test the hypotheses that subjective threat mediates the relationship between cues to action and preventive behaviours (H1) and between objective risk and preventive actions (H3), mediation analyses were performed using model number 4 (simple mediation) of the Process macro^72^ for SPSS (version 27 of the software was used). First, the analyses were conducted in the total sample; then, they were run separately in each nation (these results are described in Supplementary Materials). The bootstrapping procedure with 5000 resamples and 95% bias-corrected confidence intervals was used. Age and gender were included in each analysis as the samples differed in terms of these characteristics, and earlier studies suggested that these variables are associated with preventive behaviours.

The hypothesized moderation and moderated mediation effects were tested using the Process Macro^72^ for SPSS with 5000 bootstrap resamples. Model number 8 was applied to test H4 and H5; model number 16 was applied to test H6; model number 14 was applied to test H2 and H7. Age and gender were included as covariates. Analyses were conducted in the total sample and separately in each of the studied nations (Supplementary Materials). Variables were mean centred before creating interaction terms. All statistical tests were two-sided.

## Supplementary Information

Supplementary Information.

## Data Availability

To comply with informed participant consent, the raw Qualtrics data are protected and are not available due to data privacy. The processed, anonymized data that support the findings of this study are available from Open Science Framework: https://osf.io/vr6p4/?view_only=070d364b489b43fca34bfcfd811ed22b.

## Code availability

No custom code was used for data analysis.

## References

[1] Cucinotta D, Vanelli M (2020). WHO declares COVID-19 a pandemic. Acta Biomed..

[2] Maier BF, Brockmann D (2020). Effective containment explains subexponential growth in recent confirmed COVID-19 cases in China. Science.

[3] Michie S, West R (2021). Sustained behavior change is key to preventing and tackling future pandemics. Nat. Med..

[4] Mitsikostas DD (2020). European Headache Federation recommendations for placebo and nocebo terminology. J. Headache Pain.

[5] Kemppainen LM, Kemppainen TT, Reippainen JA, Salmenniemi ST, Vuolanto PH (2018). Use of complementary and alternative medicine in Europe: Health-related and sociodemographic determinants. Scand. J. Public Health.

[6] Linde K (2014). The use of placebo and non-specific therapies and their relation to basic professional attitudes and the use of complementary therapies among German Physicians—A cross-sectional survey. PLoS One.

[7] Asmundson GJG, Taylor S (2020). How health anxiety influences responses to viral outbreaks like COVID-19: What all decision-makers, health authorities, and health care professionals need to know. J. Anxiety Disord..

[8] Cerami C (2021). Risk-aversion for negative health outcomes may promote individual compliance to containment measures in Covid-19 pandemic. Front. Psychol..

[9] Clark C, Davila A, Regis M, Kraus S (2020). Predictors of COVID-19 voluntary compliance behaviors: An international investigation. Glob. Transit..

[10] Duan T, Jiang H, Deng X, Zhang Q, Wang F (2020). Government intervention, risk perception, and the adoption of protective action recommendations: Evidence from the COVID-19 prevention and control experience of China. Int. J. Environ. Res. Public Health.

[11] Galasso V (2020). Gender differences in COVID-19 attitudes and behavior: Panel evidence from eight countries. Proc. Natl. Acad. Sci..

[12] González-Castro JL, Ubillos-Landa S, Puente-Martínez A, Gracia-Leiva M (2021). Perceived vulnerability and severity predict adherence to COVID-19 protection measures: The mediating role of instrumental coping. Front. Psychol..

[13] Lahiri A, Jha SS, Chakraborty A, Dobe M, Dey A (2021). Role of threat and coping appraisal in protection motivation for adoption of preventive behavior during COVID-19 pandemic. Front. Public Health.

[14] Melki J (2020). Media exposure and health behavior during pandemics: The mediating effect of perceived knowledge and fear on compliance with COVID-19 prevention measures. Health Commun..

[15] Mevorach T, Cohen J, Apter A (2021). Keep calm and stay safe: The relationship between anxiety and other psychological factors, media exposure and compliance with COVID-19 regulations. Int. J. Environ. Res. Public Health.

[16] Paramita W (2021). Explaining the voluntary compliance to COVID-19 measures: An extrapolation on the gender perspective. Glob. J. Flex. Syst. Manag..

[17] Shahnazi, H. *et al. Assessing Preventive Health Behaviors from COVID-19 Based on the Health Belief Model (HBM) Among People in Golestan Province: A Cross-Sectional Study in Northern Iran*. (2020) 10.21203/rs.3.rs-24871/v1. 10.1186/s40249-020-00776-2 PMC7671178 33203453

[18] Tagini S (2021). Attachment, personality and locus of control: Psychological determinants of risk perception and preventive behaviors for COVID-19. Front. Psychol..

[19] Xu X (2021). Demographic and social correlates and indicators for behavioural compliance with personal protection among Chinese community-dwellers during COVID-19: A cross-sectional study. BMJ Open.

[20] Ufuophu-Biri E, Bebenimibo P (2021). Exploring the relationship between exposure to media messages on Covid-19 and compliance with its preventive measures among residents of Delta State, Nigeria. J. Educ. Soc. Res..

[21] Kim S, Kim S (2020). Analysis of the impact of health beliefs and resource factors on preventive behaviors against the COVID-19 pandemic. Int. J. Environ. Res. Public Health.

[22] Weston D, Ip A, Amlôt R (2020). Examining the application of behaviour change theories in the context of infectious disease outbreaks and emergency response: A review of reviews. BMC Public Health.

[23] Spadaro G, Gangl K, Prooijen J-WV, Lange PAMV, Mosso CO (2020). Enhancing feelings of security: How institutional trust promotes interpersonal trust. PLoS One.

[24] Rosenstock IM (1974). Historical origins of the health belief model. Health Educ. Monogr..

[25] Rosenstock IM, Strecher VJ, Becker MH (1988). Social learning theory and the health belief model. Health Educ. Q..

[26] Champion, V. L. & Skinner, C. S. The health belief model. In *Health Behavior and Health Education: Theory, Research, and Practice*, 4th ed. 45–65 (Jossey-Bass, 2008).

[27] Biggs, A., Brough, P. & Drummond, S. Lazarus and Folkman’s psychological stress and coping theory. In *The Handbook of Stress and Health: A Guide to Research and Practice* 351–364 (Wiley Blackwell, 2017). 10.1002/9781118993811.ch21.

[28] Lazarus, R. S. & Folkman, S. *Stress, Appraisal, and Coping*. (Springer Publishing Company, 1984).

[29] Almutairi AF, BaniMustafa A, Alessa YM, Almutairi SB, Almaleh Y (2020). Public trust and compliance with the precautionary measures against COVID-19 employed by authorities in Saudi Arabia. Risk Manag. Healthc. Policy.

[30] Ayalon L (2021). Trust and compliance with COVID-19 preventive behaviors during the pandemic. Int. J. Environ. Res. Public Health.

[31] Bargain O, Aminjonov U (2020). Trust and compliance to public health policies in times of COVID-19. J. Public Econ..

[32] Caplanova A, Sivak R, Szakadatova E (2021). Institutional trust and compliance with measures to fight COVID-19. Int. Adv. Econ. Res..

[33] Goldstein, D. A. N. & Wiedemann, J. *Who Do You Trust? The Consequences of Political and Social Trust for Public Responsiveness to COVID-19 Orders*. https://papers.ssrn.com/abstract=3580547 (2020) 10.2139/ssrn.3580547.

[34] Pak A, McBryde E, Adegboye OA (2021). Does high public trust amplify compliance with stringent COVID-19 government health guidelines? A multi-country analysis using data from 102,627 individuals. Risk Manag. Healthc. Policy.

[35] Aro AR, Vartti A-M, Schreck M, Turtiainen P, Uutela A (2009). Willingness to take travel-related health risks—A study among finnish tourists in Asia during the avian influenza outbreak. Int. J. Behav. Med..

[36] Steptoe A, Wardle J (2001). Locus of control and health behaviour revisited: A multivariate analysis of young adults from 18 countries. Br. J. Psychol..

[37] Kothari R (2021). Correlates of compliance with COVID-19 prevention guidelines: Risk propensity, locus of control, intolerance of uncertainty. Int. J. Indian Psychol..

[38] Asmundson GJG, Abramowitz JS, Richter AA, Whedon M (2010). Health anxiety: Current perspectives and future directions. Curr. Psychiatry Rep..

[39] Taylor S (2004). Understanding and treating health anxiety: A cognitive-behavioral approach. Cogn. Behav. Pract..

[40] Bavel JJV (2020). Using social and behavioural science to support COVID-19 pandemic response. Nat. Hum. Behav..

[41] Sommer, W. QAnon-ers’ magic cure for coronavirus: just drink bleach! *The Daily Beast*. https://www.thedailybeast.com/qanon-conspiracy-theorists-magic-cure-for-coronavirus-is-drinking-lethal-bleach (2020). Accessed 16 September 2021.

[42] Banai, I. P., Banai, B. & Mikloušić, I. Beliefs in COVID-19 conspiracy theories predict lower level of compliance with the preventive measures both directly and indirectly by lowering trust in government medical officials. 10.31234/osf.io/yevq7 (2020). 10.1007/s12144-021-01898-y PMC8153526 34075284

[43] Chan H-W (2021). Not-so-straightforward links between believing in COVID-19-related conspiracy theories and engaging in disease-preventive behaviours. Humanit Soc. Sci. Commun..

[44] Pummerer L (2022). Conspiracy theories and their societal effects during the COVID-19 pandemic. Soc. Psychol. Personal. Sci..

[45] Krampe H, Danbolt LJ, Haver A, Stålsett G, Schnell T (2021). Locus of control moderates the association of COVID-19 stress and general mental distress: Results of a Norwegian and a German-speaking cross-sectional survey. BMC Psychiatry.

[46] Sigurvinsdottir R, Thorisdottir IE, Gylfason HF (2020). The impact of COVID-19 on mental health: The role of locus on control and internet use. Int. J. Environ. Res. Public Health.

[47] Abalakina-Paap M, Stephan WG, Craig T, Gregory WL (1999). Beliefs in conspiracies. Polit. Psychol..

[48] Živković, A. The relationship between personality traits, locus of control and the tendency to believe in conspiracy theories. Diploma Thesis. Filozofski fakultet u Zagrebu, Department of Psychology (2018).

[49] Verlegh PWJ, Bernritter SF, Gruber V, Schartman N, Sotgiu F (2021). “Don’t Worry, We Are Here for You”: Brands as external source of control during the Covid-19 pandemic. J. Advert..

[50] Geers AL (2022). Psychosocial factors predict COVID-19 vaccine side effects. PPS.

[51] Hao F (2021). Attitudes toward COVID-19 vaccination and willingness to pay: Comparison of people with and without mental disorders in China. BJPsych Open.

[52] Le HT (2020). Feasibility of intersectoral collaboration in epidemic preparedness and response at grassroots levels in the threat of COVID-19 pandemic in Vietnam. Front. Public Health.

[53] Moehring, A. V. *et al.* Providing normative information increases intentions to accept a COVID-19 vaccine. 10.31234/osf.io/srv6t (2021). 10.1038/s41467-022-35052-4 PMC9828376 36624092

[54] Hyland-Wood B, Gardner J, Leask J, Ecker UKH (2021). Toward effective government communication strategies in the era of COVID-19. Humanit Soc. Sci. Commun..

[55] Kemmelmeier M, Jami WA (2021). Mask Wearing as Cultural Behavior: An Investigation Across 45 U.S. States During the COVID-19 Pandemic. Front. Psychol..

[56] Wang C (2021). The impact of the COVID-19 pandemic on physical and mental health in the two largest economies in the world: A comparison between the United States and China. J. Behav. Med..

[57] Henrich J, Heine SJ, Norenzayan A (2010). The weirdest people in the world?. Behav. Brain Sci..

[58] Cho H, Guo Y, Torelli C (2022). Collectivism fosters preventive behaviors to contain the spread of COVID-19: Implications for social marketing in public health. Psychol. Mark..

[59] Tei S, Fujino J (2022). Social ties, fears and bias during the COVID-19 pandemic: Fragile and flexible mindsets. Humanit Soc. Sci. Commun..

[60] Hofstede, G. *Culture’s Consequences: Comparing Values, Behaviors, Institutions and Organizations Across Nations* (SAGE Publications, 2001).

[61] Gelfand MJ (2011). Differences between tight and loose cultures: A 33-nation study. Science.

[62] Nisbett, R. E. & Cohen, D. *Culture of Honor: The Psychology of Violence in the South* (Westview Press, 1996).

[63] Huynh TLD (2020). Does culture matter social distancing under the COVID-19 pandemic?. Saf. Sci..

[64] Strain WD (2020). Development of an objective risk stratification tool to facilitate workplace assessments of healthcare workers when dealing with the CoViD-19 pandemic. medRxiv.

[65] Salkovskis PM, Rimes KA, Warwick HMC, Clark DM (2002). The Health Anxiety Inventory: Development and validation of scales for the measurement of health anxiety and hypochondriasis. Psychol. Med..

[66] Wallston KA, Strudler Wallston B, DeVellis R (1978). Development of the multidimensional health locus of control (MHLC) scales. Health Educ. Monogr..

[67] Greenglass, E., Schwarzer, R., Jakubiec, D., Fiksenbaum, L. & Taubert, S. *The Proactive Coping Inventory (PCI): A Multidimensional Research Instrument*. (1999).

[68] Cohen S, Kamarck T, Mermelstein R (1983). A global measure of perceived stress. J. Health Soc. Behav..

[69] Duncan LA, Schaller M, Park JH (2009). Perceived vulnerability to disease: Development and validation of a 15-item self-report instrument. Personal. Individ. Differ..

[70] McElroy E (2019). The CSS-12: Development and validation of a short-form version of the cyberchondria severity scale. Cyberpsychol. Behav. Soc. Netw..

[71] Kline, R. B. *Principles and Practice of Structural Equation Modeling*, 4th ed (Guilford Publications, 2015).

[72] Hayes, A. *Introduction to Mediation, Moderation, and Conditional Process Analysis* (Guilford Press, 2017).

