# Supplementary material for: Development and cross-national investigation of a model explaining participation in WHO-recommended and placebo behaviours to prevent COVID-19 infection
Source: Sci Rep. 2022 Oct 21;12:17704. doi: 10.1038/s41598-022-17303-y (PMC9586969; doi:10.1038/s41598-022-17303-y)
Supplement: Supplementary file 1 — Supplementary Information. [file 41598_2022_17303_MOESM1_ESM.docx]

**SUPPLEMENTARY MATERIALS**

**Development and cross-national investigation of a model explaining participation in WHO-recommended and placebo behaviours to prevent COVID-19 infection**

Joanna Kłosowska, Elżbieta A. Bajcar, Helena Bieniek, Justyna Brączyk, Mohsen Joshanloo, Katia Mattarozzi, Arianna Bagnis, , Moa Pontén, Maria Lalouni, Andrew L. Geers, Kelly S. Clemens, Joonha Park, Gahee Choi, Yun-Kyeung Choi, Wookyoung Jung, Eunjung Son, Hyae Young Yoon, Przemysław Bąbel

**Content:**

**Supplement A**. Detailed rationale for the study hypotheses (Note)

**Supplement B.** Demographic characteristics of the sample (Table)

**Supplement C.** Results of post-hoc tests (95% CI error bars) (Figure)

**Supplement D.** Results of mediation and moderated mediation analyses conducted separately in each of the six studied nations (Note)

**Supplement E**. Mediation analyses – Regression Coefficients (Table)

**Supplement F.** Moderation analyses – Regression Coefficients (Table)

**Supplement G.** Results of hypotheses testing in different countries – WHO actions. (Figure)

**Supplement H.** Results of hypotheses testing in different countries – placebo actions. (Figure)

**Supplement I.** Discussion of findings obtained in different nations. (Discussion)

**Supplement J**. Scales created for the purpose of the study (Methods)

**Bibliography for supplementary material**

**Supplement A. Detailed rationale for the study hypotheses**

*Health Belief Model (HBM)*

The proposed model was inspired by the Health Belief Model (HBM)^1–3^, which emphasizes the role of rational expectations in undertaking preventive actions. It includes five main constructs that are postulated to affect health behaviour^4^: perceived susceptibility (belief about the chances of getting a disease), perceived severity (belief about how serious a condition is), and a combination of them, i.e., “perceived threat”; perceived benefits (belief in the efficacy of an advised action); perceived barriers (belief about the costs of the advised action); and cues to action (factors that activate “willingness”, like media coverage).

The HBM has been successfully utilized in numerous studies analysing health behaviour predictors during the COVID-19 pandemic (e.g., ^5–8^). However, these studies usually focused on only a few selected preventive behaviours (e.g., face masks, handwashing, social distancing); in most of them, the five constructs tend to be analysed as separate and independent predictors of behaviour^9^. Meanwhile, there is evidence that the relationship between cues to action and preventive behaviours is partially mediated by perceived threat (e.g., ^10,11^). Furthermore, an interaction between perceived threat and perceived benefits and barriers to preventive actions has been hypothesized by some authors (e.g., ^4,12^).

Taking these arguments into consideration, we formulated the following hypotheses: the positive relationship between cues to action and frequency of preventive behaviours is mediated by perceived threat (H1). Decision balance moderates the relationship between perceived threat and frequency of preventive behaviours (H2). As the HBM postulates that people engage in health behaviour when the barriers to acting are outweighed by the benefits^12^ to be consistent with the theory we decided to implement a joined index that represents the difference between perceived benefits and perceived barriers (named “decision balance”) in our model.

*Integrating theories of stress and coping with HBM*

The HBM is focused on cognitive processes and does not consider the emotional component of behaviour^4^. A possible way to overcome these limitations is integration of the HBM with other models that concentrate more on emotional processes (e.g.,^13^). The theories of stress which focus on explaining people's reactions to threatening circumstances could be especially useful in explaining preventive behaviours during a pandemic.

According to Lazarus and Folkman’s^14^ transactional model of stress and coping (TMS), appraisal of the environment rather than the situation itself determines which strategies for coping with stress are initiated, if any^15,16^. This means that preventive behaviours (which are a way of coping with a threat) are the result of the subjective threat rather than the objective risk of developing serious symptoms. Thus, we hypothesized that perceived threat mediates the relationship between objective risk of developing serious COVID-19 symptoms and the frequency of preventive actions (H3).

The TMS also points out that assessment of a situation is influenced by individual beliefs^15,17^. Limited evidence has already been collected which suggests that health anxiety, i.e., the tendency to misinterpret health-related information as threatening^18,19^ is associated with attentional bias toward virus-related threatening stimuli during the COVID-19 pandemic^20^ as well as coping strategies used during lockdown^21^. Thus, we hypothesized that health anxiety moderates the relationship between objective health risk and perceived threat (H4) and the relationship between objective health risk and frequency of preventive actions (H5).

According to the TMS, people evaluate, among others, the level of control over a situation^17,22^. The locus of health control^23^, especially internal (tendency to believe that health outcomes are the effect of a person’s own behaviour), has been identified as an important correlate of pro-health behaviours^24^. On the other hand, perceiving one’s health as being influenced by others may also encourage adherence to recommendations^24^. Accordingly, we hypothesized that health locus of control (powerful internal/external others) moderates the relationship between perceived threat and the frequency of preventive behaviours (H6).

The response to threat also depends on coping style^17,22^. One type of future-oriented coping is “preventive coping”, which involves accumulation of resources to reduce the severity of potential negative outcomes of stressors^25,26^. We hypothesize that a preventive coping style moderates the relationship between perceived threat and the frequency of engaging in preventive behaviours (H7).

*Institutional trust as a predictor of preventive behaviours during the pandemic*

Institutional trust has been identified as an important predictor of engagement in preventive behaviours during the COVID-19 pandemic (e.g., ^27–37^). It may also be one of the factors responsible for the heterogeneity in compliance levels that has been observed during the pandemic between different countries^35^. Moreover, it is possible that distrust in institutions leads to increased used of placebo interventions^38,39^. Thus, we hypothesized that the frequency of WHO-recommended actions is positively associated with institutional trust (H8a), whereas the frequency of placebo actions is negatively associated with institutional trust (H8b).

| **Supplement B.** Demographic characteristics of the sample   \| Variable \| Stat. \| Italy \| Japan \| Korea \| Poland \| Sweden \| USA \| Differences \| Total sample \| \| --- \| --- \| --- \| --- \| --- \| --- \| --- \| --- \| --- \| --- \| \| Age \| Mean ±SD \| 34.17±13.14 \| 19.80±2.79 \| 43.80±14.40 \| 33.73±10.05 \| 54.05±14.20 \| 34.30±18.17 \| F(5,3340)=280.48, p<0.001, η2=0.30 \| 38.10±15.76 \| \| Sex \| N(%) \|  \| \| \| \| \| \| χ2(5)=454.81, p<0.001, Cramer’s V=0.37 \|  \| \|  \| \| Male \|  \| 118(29.14) \| 50(26.14) \| 280(50.00) \| 521(47.71) \| 86(14.88) \| 145 (27.83) \|  \| 1531(45.76) \|  \| \| Female \| 287(70.86) \| 140(73.68) \| 280(50.00) \| 571(52.29) \| 492(85.12) \| 376(72.17) \|  \| 1815(54.24) \|  \| \| Ethnicity \| N (%) \|  \|  \|  \|  \|  \|  \| χ2(25)=3188.66, p<0.001, Cramer’s V=0.44 \|  \|  \| \| White \|  \| 377(93.03) \| 9(4.74) \| 2(0.36) \| 1082(99.08) \| 565(97.75 \| 432(82.92) \|  \| 2467(73.73) \|  \| \| Black \|  \| 0(0.00) \| 0(0.00) \| 0(0.00) \| 2(0.18) \| 1(0.17) \| 24(4.61) \|  \| 27(0.81) \|  \| \| Latin \|  \| 9(2.22) \| 2(1.05) \| 0(0.00) \| 3(0.27) \| 2(0.35) \| 11(2.11) \|  \| 27(0.81) \|  \| \| Asian \|  \| 0(0.00) \| 168(88.42) \| 552(98.56) \| 1(0.09) \| 4(0.69) \| 22(4.22) \|  \| 747(22.33) \|  \| \| Arabic \|  \| 1(0.25) \| 0(0.00) \| 0(0.00) \| 1(0.09) \| 4(0.69 \| 2(0.38) \|  \| 8(0.24) \|  \| \| Mixed \|  \| 15((3.70) \| 7(3.68) \| 3(0.54) \| 1(0.09) \| 1(0.17) \| 17(3.26) \|  \| 44(1.32) \|  \| \| Other \|  \| 3(0.75 \| 4(2.11) \| 3(0.54) \| 2(0.18) \| 1(0.17) \| 13(0.54) \|  \| 26(0.78) \|  \| \| Marital Status \| N (%) \|  \|  \|  \|  \|  \|  \| χ2(30)=915.06, p<0.001, Cramer’s V=0.23 \|  \|  \| \|  \| \|  \| \| Single \|  \| 107(26.42) \| 179(94.21) \| 222(39.64) \| 263(24.08 \| 88(15.22) \| 259(49.71) \|  \| 1118(33.41) \|  \| \| Partnered \|  \| 173(42.72) \| 3(1.58) \| 4(0.71) \| 236(21.61) \| 114(19.72) \| 70(13.44 \|  \| 600(17.93) \|  \| \| Married \|  \| 100(24.69) \| 1(0.53) \| 311(55.54) \| 513(46.98) \| 280(48.44) \| 155(29.75) \|  \| 1360(40.65) \|  \| \| Divorced \|  \| 4(0.99) \| 1(0.53) \| 13(2.32) \| 31(2.84) \| 38(6.57) \| 21(4.03) \|  \| 108(3.23) \|  \| \| Separated \|  \| 9(2.22) \| 0(0.00) \| 2(0.36) \| 9(0.82) \| 22(3.81) \| 3(0.58) \|  \| 45(1.34) \|  \| \| Widowed \|  \| 7(1.73) \| 0(0.00) \| 8(1.43) \| 7(0.64) \| 22(3.81) \| 8(1.54) \|  \| 52(1.55) \|  \| \| Other \|  \| 5(1.23) \| 6(3.16) \| 0(0.00) \| 33(3.02) \| 14(2.42) \| 5(0.96) \|  \| 63(1.88) \|  \| \| Education \| N (%) \|  \|  \|  \|  \|  \|  \| χ2(25)=985.27, p<0.001, Cramer’s V=0.24 \|  \|  \| \|  \| \|  \| \|  \| \| No schooling \|  \| 1(0.25) \| 20(10.53) \| 2(0.36) \| 0(0.00) \| 0(0.00) \| 1(0.19) \|  \| 24(0.72) \|  \| \| Primary school \|  \| 0(0.00) \| 0(0.00) \| 1(0.18) \| 10(0.92) \| 0(0.00) \| 2(0.38) \|  \| 13(0.39) \|  \| \| Secondary school, \|  \| 13(3.21) \| (0)0.00 \| 5(0.89) \| 19(1.74) \| 8(1.38) \| 4(0.77) \|  \| 49(1.46) \|  \| \| High school \|  \| 196(48.40) \| 167(87.89) \| 150(26.79 \| 539(49.36) \| 84(14.53) \| 262(50.29 \|  \| 1398(41.78 \|  \| \| University graduate \|  \| 127(31.36) \| 2(1.05) \| 361(64.46 \| 480(43.96) \| 439(75.95 \| 149(28.60 \|  \| 1558(46.56 \|  \| \| Postgraduate \|  \| 68(16.79) \| 1(0.53) \| 41(7.32) \| 44(4,03) \| 47(8.13) \| 103(19.77 \|  \| 304(9.09) \|  \| \| Income (in $) \| N (%) \|  \|  \|  \|  \|  \|  \| χ2(25)=2142.77, p<0.001, Cramer’s V=0.36 \|  \|  \| \|  \| \|  \| \| < 500 \|  \| 127(31.36) \| 137(72.11) \| 108(19.29) \| 240(21.98) \| 5(0.87) \| 148(28.41) \|  \| 765(22.86) \|  \| \| 500-999 \|  \| 47(11.60) \| 43(22.63) \| 42(7.50) \| 570(52.20) \| 11(1.90) \| 59(11.32) \|  \| 772(23.07) \|  \| \| 1,000-2,499 \|  \| 175(43.21) \| 4(2.11) \| 193(34.46) \| 251(22.99) \| 114(19.72) \| 77(14.78) \|  \| 814(24.33) \|  \| \| 2,500-4,999 \|  \| 29(7.16) \| 3(1.58) \| 172(30.71) \| 22(2.01) \| 343(59.34) \| 123(23.61) \|  \| 692(20.68) \|  \| \| 5,000-9,999 \|  \| 1(0.25) \| 2(1.05) \| 42(7.50) \| 2(0.18) \| 93(16.09) \| 76(14.59) \|  \| 216(6.46) \|  \| \| 10,000 \|  \| 12(2.96) \| 0(0.00) \| 3(0.54) \| 6(0.55) \| 8(1.38) \| 35(6.72) \|  \| 64(1.91) \|  \| |
| --- | --- | --- | --- | --- | --- | --- | --- | --- | --- | --- | --- | --- | --- | --- | --- | --- | --- | --- | --- | --- | --- | --- | --- | --- | --- | --- | --- | --- | --- | --- | --- | --- | --- | --- | --- | --- | --- | --- | --- | --- | --- | --- | --- | --- | --- | --- | --- | --- | --- | --- | --- | --- | --- | --- | --- | --- | --- | --- | --- | --- | --- | --- | --- | --- | --- | --- | --- | --- | --- | --- | --- | --- | --- | --- | --- | --- | --- | --- | --- | --- | --- | --- | --- | --- | --- | --- | --- | --- | --- | --- | --- | --- | --- | --- | --- | --- | --- | --- | --- | --- | --- | --- | --- | --- | --- | --- | --- | --- | --- | --- | --- | --- | --- | --- | --- | --- | --- | --- | --- | --- | --- | --- | --- | --- | --- | --- | --- | --- | --- | --- | --- | --- | --- | --- | --- | --- | --- | --- | --- | --- | --- | --- | --- | --- | --- | --- | --- | --- | --- | --- | --- | --- | --- | --- | --- | --- | --- | --- | --- | --- | --- | --- | --- | --- | --- | --- | --- | --- | --- | --- | --- | --- | --- | --- | --- | --- | --- | --- | --- | --- | --- | --- | --- | --- | --- | --- | --- | --- | --- | --- | --- | --- | --- | --- | --- | --- | --- | --- | --- | --- | --- | --- | --- | --- | --- | --- | --- | --- | --- | --- | --- | --- | --- | --- | --- | --- | --- | --- | --- | --- | --- | --- | --- | --- | --- | --- | --- | --- | --- | --- | --- | --- | --- | --- | --- | --- | --- | --- | --- | --- | --- | --- | --- | --- | --- | --- | --- | --- | --- | --- | --- | --- | --- | --- | --- | --- | --- | --- | --- | --- | --- | --- | --- | --- | --- | --- | --- | --- | --- | --- | --- | --- | --- | --- | --- | --- | --- | --- | --- | --- | --- | --- | --- | --- | --- | --- | --- | --- | --- | --- | --- | --- | --- | --- | --- | --- | --- | --- | --- | --- | --- | --- | --- | --- | --- | --- | --- | --- | --- | --- | --- | --- | --- | --- | --- | --- | --- | --- | --- | --- | --- | --- | --- | --- | --- | --- | --- | --- | --- | --- | --- | --- | --- | --- | --- | --- | --- | --- | --- | --- | --- | --- | --- | --- | --- | --- | --- | --- | --- | --- | --- | --- | --- | --- | --- | --- | --- | --- | --- | --- | --- | --- | --- | --- | --- | --- | --- | --- | --- | --- | --- | --- | --- | --- | --- | --- | --- | --- | --- | --- | --- | --- | --- | --- | --- | --- | --- | --- | --- |

**Supplement C**. Results of post-hoc tests (95% CI error bars). *Note:* *Italy: N=405, Japan: N=190, Republic of Korea: N=551, Poland : N=1092, Sweden: N=587, USA: N=521.*


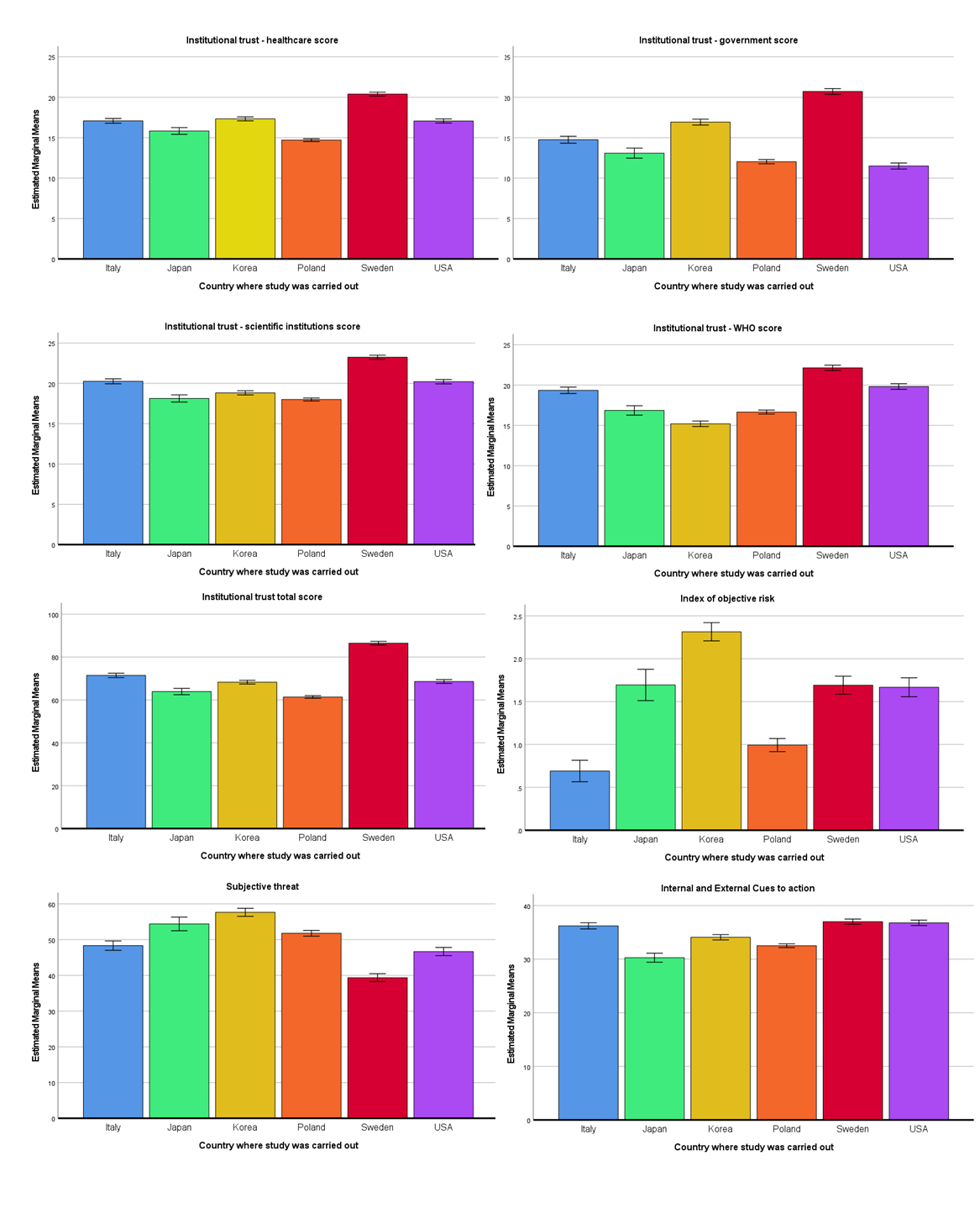


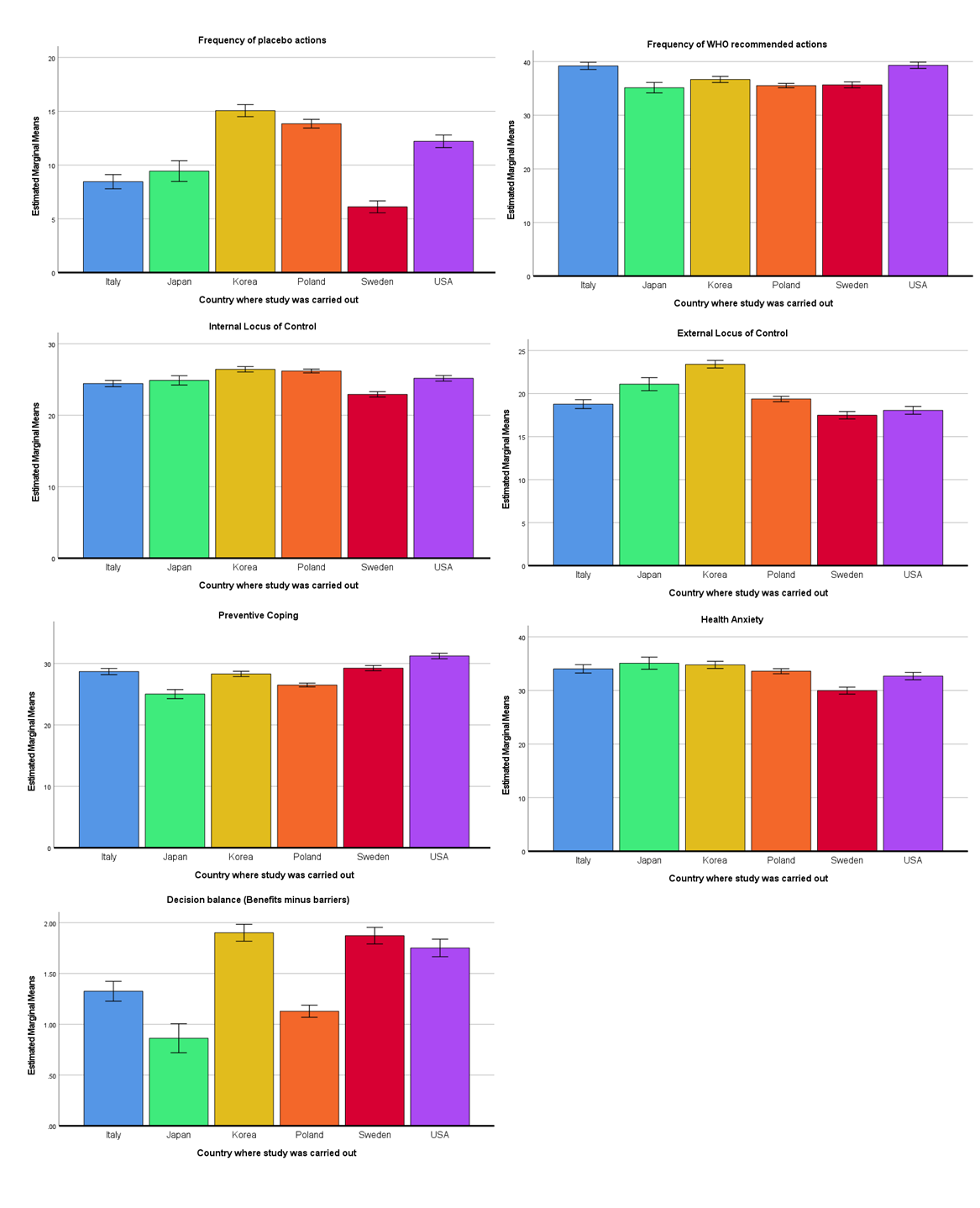


**Supplement D.** Results of mediation and moderated mediation analyses conducted separately in each of the six studied nations

*Mediation analysis*

A significant indirect effect of objective risk through subjective threat on frequency of WHO-recommended actions and placebo actions (H3) was found in Poland (WHO actions: b=0.80, SE=0.14, 95%CI: 0.55 to 1.08; placebo actions: b=0.21, SE=0.05, 95%CI: 0.11 to 0.32), Sweden (WHO actions: b=0.15, SE=0.07, 95%CI: 0.01 to 0.30; placebo actions: b=0.09, SE=0.05, 95%CI: 0.01 to 0.18), and Italy (WHO actions: b=0.26, SE=0.11, 95%CI: 0.08 to 0.50; placebo actions: b=0.13, SE=0.08, 95%CI: 0.01 to 0.31), but not in the USA, Republic of Korea and Japan. The indirect effect of cues to action on frequency of WHO-recommended actions (H1) was present in all six nations (Poland: b=0.29, SE=0.03, 95%CI: 0.24 to 0.34; Republic of Korea: b=0.05, SE=0.02, 95%CI: 0.01 to 0.10; the USA: b=0.12, SE=0.02, 95%CI: 0.08 to 0.17; Sweden: b=0.03, SE=0.01, 95%CI: 0.01 to 0.06; Italy: b=0.05, SE=0.02, 95%CI: 0.02 to 0.09; Japan: b=0.08, SE=0.04, 95%CI: 0.02 to 0.16). Furthermore, a significant indirect effect of cues to action on placebo actions was found in the USA (b=0.15, SE=0.03, 95%CI: 0.09 to 0.22), Sweden (b=0.02, SE=0.01, 95%CI: 0.00 to 0.04), and Japan (b=008, SE=0.04, 95%CI: 0.01 to 0.19).

*Moderated mediation analysis*

The interaction effect of objective risk and illness anxiety on subjective threat (H4) was found only in the USA (b=-0.12, SE=0.03, 95%CI: -0.18 to -0.05, p<0.01). The relationship between objective risk and subjective threat was positive and significant only among Americans with low (mean-1SD) levels of health anxiety (Figure 3). Also, the mediation effect of objective risk through subjective threat was stronger among individuals with low levels of health anxiety than among those characterized by higher levels (index of moderated mediation=-0.01, SE=0.01, 95%CI: -0.03 to -0.01).

The moderation effect of illness anxiety on the relationship between objective risk and preventive actions (H5) was confirmed in two of the examined nations, but only in the case of placebo actions: USA (b=-0.12, SE=0.02, 95%CI: -0.18 to -0.05, p<0.001) and Japan (b=-0.30, SE=0.13, 95%CI: -0.56 to -0.04, p<0.05). In Japan, the positive relationship between objective risk and frequency of placebo actions was stronger for individuals characterized by low levels of health anxiety than those characterized by higher levels. The opposite pattern was found in the USA (Figure 3).

The interaction effect of subjective threat and locus of health control (H6) was confirmed in Poland, Republic of Korea, Sweden and USA. In Poland, the relationship between subjective threat and both types of preventive actions was moderated by the external locus of health control (WHO-recommended actions: b=-0.01, SE=0.00, 95%CI: -0.02 to -0.01, p<0.001; placebo actions: b=0.01, SE=0.00, 95%CI: 0.00 to 0.01, p<0.05). The effect of subjective threat on the frequency of WHO-recommended actions was reduced in the case of individuals with high levels of external LoC (Figure 4). Additionally, the mediation effect of objective risk through subjective threat on WHO actions was significant only in participants who reported low levels of external LoC (index of moderated mediation: b=-0.03, SE=0.01, 95%CI: -0.05 to -0.01) but not in those who reported medium or high levels. On the other hand, the positive effect of subjective threat on placebo actions was reduced among participants with low levels of external LoC, and the indirect effect of perceived threat on placebo actions was significant only among individuals who reported high external LoC (index of moderated mediation=0.01, SE=0.01, 95%CI: 0.00 to 0.03).

Similar results were obtained in the USA: the interaction effect of subjective threat and external LoC on placebo actions was significant (b=0.02, SE=0.00, 95%CI: 0.01 to 0.02, p<0.001); the relationship between feeling threatened and the frequency of applying placebo interventions was positive and was stronger in individuals characterized by high levels of external LoC than in individuals characterized by medium or low levels (Figure 4). However, the indirect effect of objective risk through perceived threat was not moderated by external LoC (index of moderated mediation: b=0.01, SE=0.01, 95%CI: -0.02 to 0.03).

A different pattern of results was obtained in the Republic of Korea and Sweden. In these nations, there was a significant interaction effect of subjective threat and internal LoC on frequency of placebo actions (Republic of Korea: b=0.02, SE=0.01, 95%CI: 0.01 to 0.03, p<0.01; Sweden: b=-0.01, SE=0.00, 95%CI: -0.02 to -0.00, p<0.05). Interestingly, in Sweden, a stronger positive effect of subjective threat on frequency of placebo actions was observed among individuals with low levels of internal LoC than in those with medium and high levels. Nonetheless, the mediation effect of objective risk through subjective threat was not moderated by internal LoC (index of moderated mediation=-0.01, SE=0.01, 95%CI: -0.03 to 0.00). In the Republic of Korea, the positive effect of perceived threat was stronger among people with higher levels of internal LoC than among people with lower levels (Figure 4). Similarly, as in Sweden, the indirect effect of objective risk was not moderated by internal LoC (index of moderated mediation=0.02, SE=0.02, 95%CI: -0.02 to 0.06).

The interaction effect of subjective threat and preventive coping style on preventive behaviours (H7) was confirmed only in Poland, and only for placebo actions (b=0.01, SE=0.00, 95%CI: 0.00 to 0.01, p<0.05). The positive relationship between subjective threat and frequency of engaging in placebo actions was stronger among individuals characterized by high levels of preventive coping style than in individuals characterized by lower levels (Figure 3). Indirect effect objective risk through subjective threat did not vary at the level of the moderator variable (index of moderated mediation=0.01, SE=0.01, 95%CI: -0.00 to 0.03).

The interaction effect of subjective threat and decision balance (H2) was present in all of the analysed nations. However, in most cases this effect was confirmed only for one type of preventive actions and not the other. The only exception was Poland, where a significant interaction effect was found for both WHO-recommended actions (b=-0.03, SE=0.01, 95%CI: -0.05 to -0.01, p<0.01) and placebo actions (b=-0.03, SE=0.01, 95%CI: -0.05 to -0.01, p<0.01). A reduced positive effect of perceived threat on frequency of both types of behaviours was observed among individuals who perceived more benefits than barriers associated with preventive actions (Figure 5). In the case of WHO-recommended actions, a similar effect was observed in Japan (b=-0.06, SE=0.03, 95%CI: -0.11 to -0.00, p<0.05), Italy (b=-0.07, SE=0.02, 95%CI: -0.12 to -0.03, p<0.001), Sweden (b=-0,05, SE=0.02, 95%CI: -0.09 to -0.01, p<0.05) and the Republic of Korea (b=-0.08, SE=0.01, 95%CI: -0.11 to -0.05, p<0.001). In the USA, this effect was found only in the case of placebo actions (b=-0.08, SE=0.02, 95%CI: -0.12 to -0.05, p<0.001). Additionally, in Poland, Sweden and Italy, the indirect effect of objective risk through subjective threat on WHO-recommended actions was stronger for participants who declared they perceived more barriers than benefits of actions than participants who perceived more benefits than barriers (index of moderated mediation= Poland:-0.09, SE=0.04, 95%CI: -0.18 to -0.01; Sweden: -0.05, SE=0.03, 95%CI: -0.12 to -0.00; Italy: b=-0.17, SE=0.08, 95%CI: -0.35 to -0.04). In Poland, a similar pattern was also found in the case of placebo actions (index of moderated mediation=-0.08, SE=0.04, 95%CI: -0.16 to -0.01) (see Table 4 and Supplement G, H for summary).

**Supplement E.** Mediation analyses – Regression Coefficients

| Pd | | Parameter | | Total sample  (95%CI) | Italy  (95%CI) | Japan  (95%CI) | Korea  (95%CI) | Poland  (95%CI) | Sweden  (95%CI) | USA  (95%CI) |
| --- | --- | --- | --- | --- | --- | --- | --- | --- | --- | --- |
| Objective risk | WHO actions | | |  |  |  |  |  |  |  |
|  |  | | Total effect | 0.441(0.203, 0.678) | -0.231(-0.991, 0.528) | -0.138(-3.178, 2.902) | -0.094(-0.706, 0.518) | 0.839(0.316, 1.362) | 0.570(0.080, 1.061) | 0.376(-0.133,0.884) |
|  |  | | Direct effect | 0.002(-0.223, 0.227) | -0.487(-1.236, 0.262) | 0.391(-2.521, 3.303) | -0.172(-0.761, 0.417) | 0.036(-0.420, 0.493) | 0.420(-0.052, 0.891) | 0.329(-0.145, 0.802) |
|  |  | | Indirect effect | 0.439(0.338, 0.538) | 0.256(0.081, 0.502) | -0.529(-1.673, 0.538) | 0.078(-0.101, 0.274) | 0.803(0.545,1.081) | 0.151(0.014, 0.300) | 0.047(-0.137, 0.247) |
|  |  | | Pd🡪M | 2.483(1.995, 2.970) | 2.284(0.752, 3,816) | -3.599(-9.926, 2.729) | 0.645(-0.741, 2.031) | 2.714(1.810, 3,618) | 1.070(0.056, 2.084) | 0.308(-0.910, 1.526) |
|  |  | | M🡪DV | 0.177(0.161, 0.192) | 0.112(0.064, 0.159) | 0.147(0.081, 0.213) | 0.121(0.086, 0.157) | 0.296(0.266, 0.325) | 0.141(0.103, 0.179) | 0.153(0.119, 0.186) |
|  |  | | R^2^ | 0.138 | 0.108 | 0.095 | 0.112 | 0.292 | 0.134 | 0.203 |
|  |  | | F | F(4,3341)=134.692^***^ | F(4,400)=12.137^***^ | F(4,185)=4.834^**^ | F(4,555)=17.469^***^ | F(4,1087)=112.096^***^ | F(4,573)=22.222^***^ | F(4,516)=32.879^***^ |
|  | Placebo actions | | |  |  |  |  |  |  |  |
|  |  | | Total effect | 0.802(0.594, 1.055) | 0.814(-0.048, 1.676) | 0.035(-3.570, 3.640) | -0.459(-1.159, 0.241) | 0.976(0.538, 1.413) | 0.241(-0.165, 0.647) | -0.377(-1.093,0.338) |
|  |  | | Direct effect | 0.404(0.160, 0.648) | 0.685(-0.183, 1.554) | 0.516(-3.011, 4.042) | -0.518(-1.208, 0.171) | 0.771(0.332, 1.211) | 0.155(-0.245, 0.554) | -0.441(-1.111, 0.230) |
|  |  | | Indirect effect | 0.399(0.312, 0.494) | 0.129(0.006, 0.307) | -0.481(-1.551, 0.534) | 0.059(-0.072, 0.230) | 0.205(0.108, 0.319) | 0.086(0.007, 0.184) | 0.063(-0.205, 0.314) |
|  |  | | Pd🡪M | 2.483(1.995, 2.970) | 2.284(0.752, 3.816) | -3.599(-9.926, 2.729) | 0.645(-0.741, 2.031) | 2.714(1.810, 3.618) | 1.070(0.056, 2.084) | 0.308(-0.910, 1.526) |
|  |  | | M🡪DV | 0.161(0.144, 0.177) | 0.056(0.001, 0.111) | 0.134(0.053, 0.214) | 0.092(0.050,0.133) | 0.075(0.047, 0.104) | 0.081(0.048, 0.113) | 0.206(0.159, 0.254) |
|  |  | | R^2^ | 0.109 | 0.049 | 0.069 | 0.060 | 0.053 | 0.081 | 0.128 |
|  |  | | F | F(4,3341)=101.912^***^ | F(4,400)=5.215^***^ | F(4, 185)=3.438^**^ | F(4,555)=8.923^***^ | F(4,1087)=15.344^***^ | F(4,573)=12.763^***^ | F(4,516)=18.975^***^ |
| Cues to action | WHO actions | | |  |  |  |  |  |  |  |
|  |  | | Total effect | 0.424(0.389, 0.459) | 0.234(0.129, 0.339) | 0.321(0.153, 0.490) | 0.390(0.317, 0.463) | 0.534(0.468, 0.601) | 0.237(0.144, 0.330) | 0.339(0.259, 0.419) |
|  |  | | Direct effect | 0.332(0.297, 0.368) | 0.186(0.079, 0.293) | 0.242(0.072, 0.411) | 0.339(0.287, 0.419) | 0.245(0.169, 0.321) | 0.205(0.115, 0.295) | 0.219(0.132, 0.306) |
|  |  | | Indirect effect | 0.092(0.077, 0.107) | 0.049(0.019, 0.087) | 0.080(0.024, 0.163) | 0.051(0.009, 0.095) | 0.290(0.234, 0.343) | 0.032(0.007, 0.060) | 0.120(0.077, 0.168) |
|  |  | | Pd🡪M | 0.676(0.600, 0.752) | 0.561(0.348, 0.773) | 0.655(0.301, 1.008) | 0.925(0.761, 1.090) | 1.252(1.146, 1.358) | 0.239(0.043, 0.434) | 1.087(0.907, 1.267) |
|  |  | | M 🡪 DV | 0.135(0.120, 0.151) | 0.087(0.039, 0.134) | 0.122(0.054, 0.189) | 0.055(0.018, 0.092) | 0.232(0.197, 0.267) | 0.135(0.098, 0.173) | 0.110(0.073, 0.147) |
|  |  | | R^2^ | 0.219 | 0.130 | 0.131 | 0.209 | 0.317 | 0.159 | 0.236 |
|  |  | | F | F(4,3341)=233.841^***^ | F(4,400)=14.928^***^ | F(4,185)=7.000^***^ | F(4,555)=36.703^***^ | F(4,1087)=126.134^***^ | F(4,573)=27.116^***^ | F(4,516)=39.896^***^ |
|  | Placebo actions | | |  |  |  |  |  |  |  |
|  |  | | Total effect | 0.151(0.110, 0.191) | 0.148(0.027, 0.270) | 0.179(-0.028, 0.385) | 0.345(0.262, 0.436) | 0.238(0.177, 0.299) | 0.119(0.041, 0.197) | 0.480(0.368, 0.592) |
|  |  | | Direct effect | 0.043(0.002, 0.083) | 0.121(-0.005, 0.246) | 0.098(-0.111, 0.307) | 0.323(0.227, 0.419) | 0.198(0.124, 0.272) | 0.100(0.024, 0.177) | 0.327(0.204, 0.450) |
|  |  | | Indirect effect | 0.108(0.091, 0.126) | 0.027(-0.004, 0.062) | 0.080(0.014, 0.185) | 0.026(-0.023, 0.074) | 0.040(-0.005, 0.085) | 0.019(0.004, 0.039) | 0.154(0.093, 0.221) |
|  |  | | Pd🡪M | 0.676(0.600, 0.752) | 0.561(0.348, 0.773) | 0.655(0.301, 1.008) | 0.925(0.761, 1.090) | 1.252(1.146, 1.358) | 0.239(0.043, 0.434) | 1.087(0.907, 1.267) |
|  |  | | M🡪DV | 0.160(0.143, 0.177) | 0.049(-0.007, 0.105) | 0.123(0.040, 0.205) | 0.028(-0.017, 0.072) | 0.032(-0.002, 0.066) | 0.078(0.046, 0.110) | 0.141(0.089, 0.194) |
|  |  | | R^2^ | 0.107 | 0.052 | 0.073 | 0.126 | 0.067 | 0.091 | 0.169 |
|  |  | | F | F(4,3341)=100.177^***^ | F(4,400)=5.522^***^ | F(4,185)=3.646^**^ | F(4,555)=19.913^***^ | F(4,1087)=19.446^***^ | F(4,573)=14.394^***^ | F(4,516)=26.266^***^ |

*Note:* **p<0.05, **p<0.01, ***p<0.001, Pd-predictor, M-mediator (Subjective threat), DV-dependent variable. Gender and age were controlled for. Total sample: N=3346; Italy: N=405, Japan: N=190, Republic of Korea: N=551, Poland : N=1092, Sweden: N=587, USA: N=521.*

**Supplement F.** Moderation analyses – Regression Coefficients

| H | Parameter | | Total sample  (95%CI) | Italy  (95%CI) | Japan  (95%CI) | Korea  (95%CI) | Poland  (95%CI) | Sweden  (95%CI) | USA  (95%CI) |
| --- | --- | --- | --- | --- | --- | --- | --- | --- | --- |
| Hypothesis 4 and 5 | Subjective threat | |  |  |  |  |  |  |  |
|  |  | Objective risk (1) | 1.942 (1.489, 2.394) | 1.459 (0.005, 2.912) | -2.138 (-8.403, 4.126) | -0.153 (-1.468, 1.162) | 1.930 (1.016, 2.844) | 0.594 (-0.341,1.528) | 0.878(-0.206,1.963) |
|  |  | Health anxiety (2) | 0.687 (0.631, 0.744) | 0.608 (0.446, 0.769) | 0.422 (0.149, 0.694) | 0.568 (0.442, 0.694) | 0.508 (0.405, 0.611) | 0.719(0.585, 0.854) | 0.701(0.593,0.808) |
|  |  | Interaction: (1×2) | 0.001 (-0.035, 0.038) | -0.131 (-0.322, 0.060) | -0.086 (-0.0562, 0.390) | 0.028 (-0.062, 0.118) | -0.003 (-0.085, 0.080) | 0.029(-0.057, 0.115) | -0.118(-0.181,-0.054) |
|  |  | R^2^ | 0.180 | 0.248 | 0.073 | 0.144 | 0.136 | 0.190 | 0.276 |
|  |  | F | F(5,3340)=146.46^***^ | F (5, 399)=26.289^***^ | F(5, 184)=2.925^*^ | F(5, 554)=18.618^***^ | F(5,1086)=34.271^***^ | F(5,572)=26.873^***^ | F(5,515)=32.321^***^ |
|  |  | ∆R^2^ | 0.000 | 0.004 | 0.001 | 0.001 | 0.000 | 0.001 | 0.019 |
|  | WHO actions | |  |  |  |  |  |  |  |
|  |  | Objective risk (1) | 0.024 (-0.200, 0.248) | -0.434 (-1.189, 0.320) | 0.703 (-2.178, 3.585) | -0.116 (-0.713, 0.482) | 0.221 (-0.249, 0.692) | 0.490 (0.024, 0.956) | 0.287(-0.199,0.773) |
|  |  | Health anxiety (2) | -0.096 (-0.126,-0.066) | -0.050 (-0.139, 0.039) | 0.181 (0.053, 0.310) | -0.026 (-0.087, 0.035) | -0.160(-0.215, -0.105) | -0.152(-0.226,-0.079) | -0.082(-0.137,-0.027) |
|  |  | Interaction (1×2) | -0.008 (-0.025, 0.010) | 0.024 (-0.075, 0.123) | 0.197 (-0.022, 0.415) | -0.015 (-0.056, 0.026) | -0.002 (-0.044, 0.041) | -0.022(-0.064,0.021) | 0.024(-0.005,0.053) |
|  |  | Subjective threat | 0.197 (0.181, 0.214) | 0.123 (0.072, 0.174) | 0.129 (0.062, 0.196) | 0.128 (0.090, 0.166) | 0.321 (0.291, 0.352) | 0.175(0.134,0.216) | 0.185(0.146,223) |
|  |  | R^2^ | 0.149 | 0.11 | 0.139 | 0.114 | 0.313 | 0.160 | 0.219 |
|  |  | F | F(6, 3339)=97.711 ^***^ | F (6, 398)=8.329^***^ | F(6, 183)=4.941^***^ | F (6, 553)=11.858^***^ | F(6,1085)=82.440^***^ | F(6,571)=18.097^***^ | F(6,514)=24.049^***^ |
|  |  | ∆R^2^ | 0.000 | 0.001 | 0.015 | 0.001 | 0.000 | 0.002 | 0.004 |
|  | Placebo actions | |  |  |  |  |  |  |  |
|  |  | Objective risk (1) | 0.365 (0.123, 0.608) | 0.669 (-0.204, 1.541) | 1.456 (-1.971, 4.883) | -0.603 (-1.296, 0.090) | 0.641 (0.183, 1.098) | 0.144(-0.257,0.544) | -0.524(-1.184,0.136) |
|  |  | Health anxiety (2) | 0.117 (0.085, 0.150) | 0.090 (-0.13, 0.192) | 0.227 (0.074, 0.379) | 0.111 (0.040, 0.182) | 0.080 (0.027, 0.134) | 0.024(-0.039,0.087) | 0.197(0.122,0.272) |
|  |  | Interaction (1×2) | -0.023 (-0.042,-0.003) | 0.071 (-0.044, 0.185) | -0.302 (-0.562, -0.042) | -0.027 (-0.074, 0.021) | 0.013(-0.028, 0.054) | 0.003(-0.034,0.040) | -0.112(-0.151,-0.073) |
|  |  | Subjective threat | 0.136 (-0.042, -0.003) | 0.041 (-0.018, 0.100) | 0.100 (0.021, 0.180) | 0.068 (0.024, 0.112) | 0.062(0.033, 0.092) | 0.075(0.040,0.110) | 0.120(0.067,0.173) |
|  |  | R^2^ | 0.123 | 0.060 | 0.147 | 0.077 | 0.062 | 0.083 | 0.212 |
|  |  | F | F(6, 3339)=77.843^***^ | F(6, 398)=4.200^***^ | F(6,183)=5.233^***^ | F(6, 553)=7.731^***^ | F(6, 1085)=11.92^***^ | F(6,571)=8.586^***^ | F(6,514)=23.105^***^ |
|  |  | ∆R^2^ | 0.001^*^ | 0.004 | 0.025^*^ | 0.002 | 0.000 | 0.000 | 0.049^***^ |
| Hypothesis 6 | WHO actions | |  |  |  |  |  |  |  |
|  |  | Subjective threat (1) | 0.167(0.150,0.183) | 0.107(0.058,0.156) | 0.126(0.059,0.193) | 0.083(0.048,0.118) | 0.272(0.242,0.303) | 0.142(0.104,0.180) | 0.153(0.116,0.191) |
|  |  | Internal LoC (2) | 0.160(0.111,0.209) | 0.145(0.025,0.265) | 0.266(0.066,0.466) | 0.335(0.214,0.456) | 0.281(0.191,0.371) | 0.115(0.010,0.219) | 0.112(0.008,0.216) |
|  |  | External LoC (3) | 0.015(-0.029,0.058) | 0.080(-0.034,0.193) | 0.120(-0.101,0.340) | 0.209(0.093,0.326) | 0.110(0.034,0.185) | 0.007(-0.088,0.102) | 0.015(-0.082,0.113) |
|  |  | Interaction (1×2) | 0.004(0.001,0.007) | -0.007(-0.018, 0.004) | 0.008(-0.005,0.021) | 0.004(-0.005,0.014) | 0.002(-0.004,0.007) | -0.008(-0.017,-0.001) | -0.003(-0.010, 0.003) |
|  |  | Interaction (1×3) | -0.008(-0.010,-0.005) | 0.001(-0.008, 0.010) | -0.007(-0.022,0.008) | 0.003(-0.005,0.011) | -0.012(-0.016,-0.008) | -0.006(-0.014,0.002) | -0.004(-0.010,0.001) |
|  |  | Objective risk | -0.020(-0.245,0.205) | -0.627(-1.379,0.125) | 0.491(-2.378,3.360) | -0.372(-0.932,0.188) | 0.071(-0.374,0.517) | 0.417(-0.058,0.892) | 0.317(-0.165,0.800) |
|  |  | R^2^ | 0.156 | 0.132 | 0.149 | 0.211 | 0.338 | 0.151 | 0.223 |
|  |  | F | F(8,3337)=77.181^***^ | F(8,396)=7.527^***^ | F(8,181)=3.965^***^ | F(8,551)=18.402^***^ | F(8,1083)=69.093^***^ | F(8,569)=12.660^***^ | F(8,512)=18.327 |
|  |  | ∆R^2^(1×2) | 0.001^*^ | 0.004 | 0.007 | 0.001 | 0.000 | 0.004 | 0.002 |
|  |  | ∆R^2^(1×3) | 0.008^***^ | 0.000 | 0.004 | 0.001 | 0.020^***^ | 0.003 | 0.004 |
|  | Placebo actions | |  |  |  |  |  |  |  |
|  |  | Subjective threat (1) | 0.119(0.101,0.136) | 0.050(-0.008, 0.108) | 0.121(0.039,0.204) | 0.051(0.009,0.093) | 0.035(0.006,0.065) | 0.082(0.050,0.115) | 0.118(0.068,0.168) |
|  |  | Internal LoC (2) | 0.132(0.080,0.184) | 0.053(-0.088, 0.194) | -0.167(-0.412,0.079) | 0.076(-0.069,0.220) | -0.005(-0.091,0.082) | 0.121(0.033,0.209) | -0.003(-0.141,0.135) |
|  |  | External LoC (3) | 0.252(0.206,0.299) | 0.050(-0.083,0.183) | 0.299(0.029,0.570) | 0.317(0.179,0.456) | 0.296(0.224,0.369) | -0.007(-0.087,0.073) | 0.397(0.268,0.526) |
|  |  | Interaction (1×2) | 0.000(-0.003,0.004) | 0.000(-0.013, 0.013) | 0.004(-0.012,0.20) | 0.018(0.006, 0.029) | 0.002(-0.004,0.007) | -0.008(-0.016,-0.001) | 0.008(-0.001,0.017) |
|  |  | Interaction (1×3) | 0.008(0.005,0.011) | -0.001(-0.11,0.010) | 0.002(-0.017,0.021) | 0.004(-0.006,0.013) | 0.005(0.001, 0.009) | -0.006(-0.013,0.001) | 0.016(0.009,0.024) |
|  |  | Objective risk | 0.261(0.022,0.500) | 0.050(-0.008,0.108) | 0.145(-3.376,3.666) | -0.670(-1.337,-0.003) | 0.585(0.156,1.014) | 0.165(-0.236,0.565) | -0.018(-0.658,0.623) |
|  |  | R^2^ | 0.164 | 0.053 | 0.101 | 0.135 | 0.114 | 0.108 | 0.253 |
|  |  | F | F(8,3337)=81.780^***^ | F(8,396)=2.771^**^ | F(8,181)=2.549^*^ | F(8,551)=10.765 | F(8,1083)=17.364^***^ | F(8,569)=8.625^***^ | F(8,512)=21.620^***^ |
|  |  | ∆R^2^(1×2) | 0.000 | 0.000 | 0.001 | 0.014^**^ | 0.000 | 0.007* | 0.005 |
|  |  | ∆R^2^(1×3) | 0.008^***^ | 0.000 | 0.000 | 0.001 | 0.004^*^ | 0.004 | 0.027^***^ |
| Hypothesis 7 | WHO actions | |  |  |  |  |  |  |  |
|  |  | Subjective threat (1) | 0.171(0.156, 0.186) | 0.103(0.056, 0.150) | 0.119(0.051,0.188) | 0.107(0.072,0.141) | 0.281(0.252, 0.310) | 0.129(0.092,0.167) | 0.149(0.115, 0.182) |
|  |  | Preventive coping (2) | 0.316(0.277,0.355) | 0.237(0.118,0.356) | 0.248(0.083,0.412) | 0.310(0.221,0.398) | 0.312(0.231,0.393) | 0.214(0.125,0.303) | 0.136(0.059,0.213) |
|  |  | Interaction (1×2) | -0.002(-0.005,0.001) | 0.001(-0.008,0.010) | 0.003(-0.007,0.013) | -0.003(-0.010,0.003) | -0.002(-0.007,0.002) | -0.002(-0.010,0.006) | -0.001(-0.006,0.004) |
|  |  | Objective risk | 0.072(-0.145,0.289) | -0.466(-1.205, 0.274) | 0.322(-2.559,3.203) | -0.168(-0.734,0.399) | 0.048(-0.397,0.492) | 0.551(0.085,1.016) | 0.420(-0.053,0.892) |
|  |  | R^2^ | 0.202 | 0.142 | 0.139 | 0.182 | 0.329 | 0.168 | 0.223 |
|  |  | F | F(6, 3339)=140.573 | F(6, 398)=11.000^***^ | F(6,183)=4.932^***^ | F(6,553)=20.546^***^ | F(6,1085)=88.779^***^ | F(6,571)=19.272 | F(6,514)=24.609^***^ |
|  |  | ∆R^2^(1×2) | 0.001 | 0.000 | 0.001 | 0.002 | 0.001 | 0.000 | 0.000 |
|  | Placebo actions | |  |  |  |  |  |  |  |
|  |  | Subjective threat (1) | 0.157(0.141,0.174) | 0.057(0.001,0.112) | 0.081(0.03, 0.160) | 0.072(0.032,0.112) | 0.067(0.038,0.95) | 0.079(0.047,0.111) | 0.202(0.155,0.250) |
|  |  | Preventive coping (2) | 0.952(0.051, 0.139) | 0.001(-0.139,0.142) | 0.478(0.288,0.668) | 0.407(0.304,0.509) | 0.155(0.076,0.234) | 0.030(-0.047,0.107) | 0.018(-0.092,0.128) |
|  |  | Interaction (1×2) | 0.008(0.005, 0.011) | 0.003(-0.008,0.013) | 0.008(-0.004,0.019) | -0.002(-0.009,0.005) | 0.005(0.000,0.009) | -0.004(-0.010, 0.003) | 0.007(-0.000,0.014) |
|  |  | Objective risk | 0.422(0.179, 0.664) | 0.663(-0.212,1.537) | 0.465(-2.871,3.801) | -0.516(-1.170,0.140) | 0.776(0.340,1.212) | 0.174(-0.228,0.576) | -0.404(-1.079,0.271) |
|  |  | R^2^ | 0.122 | 0.050 | 0.191 | 0.154 | 0.069 | 0.085 | 0.134 |
|  |  | F | F(6,3339)=77.089^***^ | F(6,398)=3.505^**^ | F(6,183)=7.192^***^ | F(6,553)=16.795^***^ | F(6,1085)=13.306^***^ | F(6,571)=8.846^***^ | F(6,514)=13.295^***^ |
|  |  | ∆R^2^(1×2) | 0.009^***^ | 0.001 | 0.007 | 0.000 | 0.004^*^ | 0.002 | 0.006 |
| Hypothesis 8 | WHO actions | |  |  |  |  |  |  |  |
|  |  | Subjective threat (1) | 0.173(0.159, 0.187) | 0.057(0.002,0.112) | 0.197(0.128,0.266) | 0.133(0.101,0.165) | 0.257(0.230,0.285) | 0.167(0.130,0.204) | 0.130(0.098,0.162) |
|  |  | Benefits minus barriers (2) | 2.228(2.034,2.422) | -0.413(-1.050,0.224) | 1.384(0.486,2.281) | 1.888(1.495,2.281) | 2.693(2.305,3.081) | 1.779(1.279,2.280) | 2.055(1.634,2.475) |
|  |  | Interaction (1×2) | -0.048(-0.059,-0.036) | 0.027(-0.025,0.078) | -0.057(-0.112,-0.001) | -0.079(-0.106,-0.052) | -0.033(-0.054,-0.011) | -0.046(-0.086,-0.007) | -0.020(-0.045,0.005) |
|  |  | Objective risk | -0.114(-0.322,0.093) | 0.703(-0.165,1.570) | -0.007(-2.829,2.816) | -0.079(-0.612,0.455) | 0.230(-0.189,0.648) | 0.541(0.088,0.994) | -0.077(-0.524,0.370) |
|  |  | R^2^ | 0.270 | 0.056 | 0.167 | 0.278 | 0.409 | 0.207 | 0.330 |
|  |  | F | F(6, 3339)=206.056^***^ | F(6,398)=3.951^***^ | F(6,183)=6.111^***^ | F(6,553)=35.512^***^ | F(6,1085)=125.267^***^ | F(6,571)=24.796^***^ | F(6,514)=42.155^***^ |
|  |  | ∆R^2^(1×2) | 0.014^***^ | 0.002 | 0.018^*^ | 0.043^***^ | 0.05^**^ | 0.007^*^ | 0.003 |
|  | Placebo actions | |  |  |  |  |  |  |  |
|  |  | Subjective threat (1) | 0.161(0.144,0.178) | 0.111(0.065,0.156) | 0.126(0.039,0.212) | 0.095(0.054,0.137) | 0.077(0.048,0.106) | 0.091(0.058,0.123) | 0.196(0.149,0.242) |
|  |  | Benefits minus barriers (2) | -0.706(-0.934,-0.479) | 1.343(0.817,1.870) | -0.652(-1.780,0.476) | -0.653(-1.159,-0.147) | -0.459(-0.866,-0.052) | 0.701(0.262,1.140) | -2.233(-2.844,-1.623) |
|  |  | Interaction (1×2) | -0.004(-0.018,0.010) | -0.074(-0.117,-0.032) | -0.021(-0.091,0.049) | -0.028(-0.063,0.006) | -0.030(-0.053,-0.008) | -0.016(-0.051,0.018) | -0.083(-0.119,-0.046) |
|  |  | Objective risk | 0.439(0.196, 0.682) | -0.537(-1.254,0.180) | 0.460(-3.089,4.009) | -0.481(-1.168,0.205) | 0.739(0.300,1.178) | 0.203(-0.195,0.601) | 0.171(-0.478,0.820) |
|  |  | R^2^ | 0.119 | 0.188 | 0.077 | 0.075 | 0.062 | 0.098 | 0.229 |
|  |  | F | F(6, 3339)=74.832^***^ | F(6,398)=15.327^***^ | F(6,183)=2.528^*^ | F(6,553)=7.460^***^ | F(6,1085)=12.005 ^***^ | F(6,571)=10.359^***^ | F(6,514)=25.424^***^ |
|  |  | ∆R^2^(1×2) | 0.000 | 0.024^***^ | 0.002 | 0.004 | 0.006^**^ | 0.001 | 0.030^***^ |

*Note:* **p<0.05, **p<0.01, ***p<0.001, H – hypothesis. Gender and age were controlled for. Total sample: N=3346; Italy: N=405, Japan: N=190, Republic of Korea: N=551, Poland : N=1092, Sweden: N=587, USA: N=521.*

**Supplement G.** Results of hypotheses testing in different countries – WHO actions. *Note: The hypotheses confirmed in the total sample are marked with asterisks. Flags represent the countries for which a given hypothesis was confirmed.*


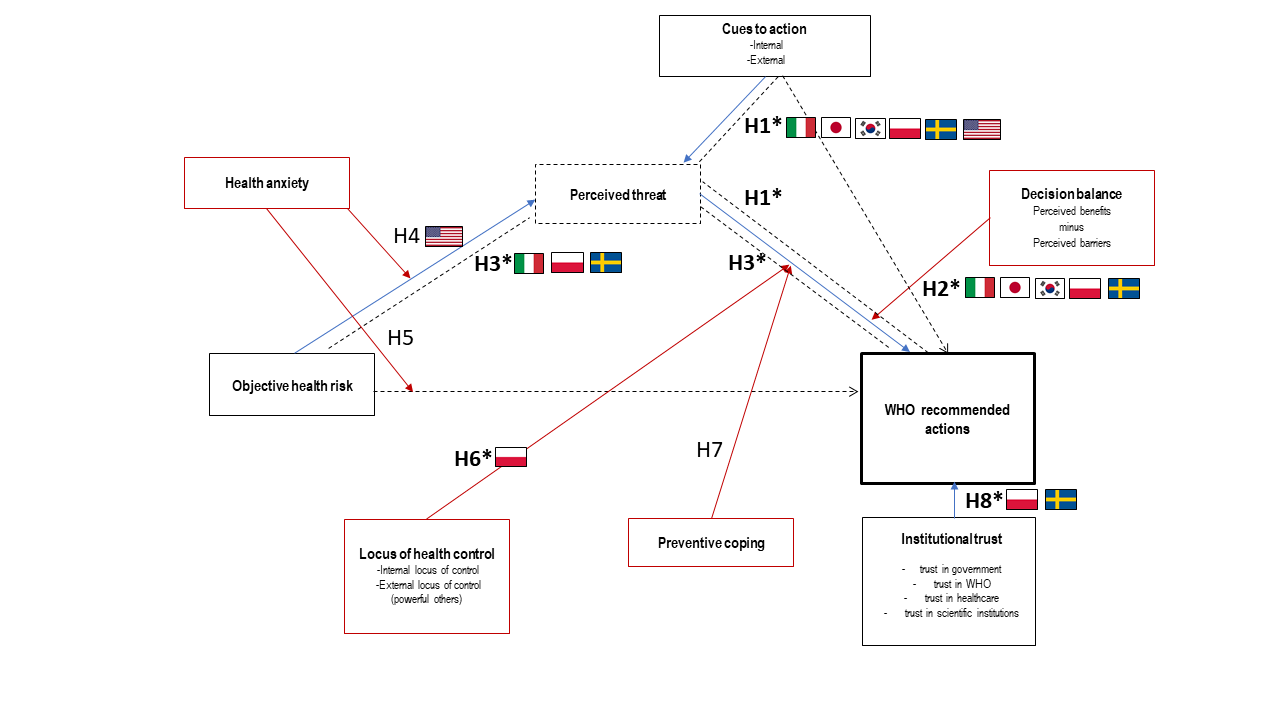


**Supplement H.** Results of hypotheses testing in different countries – Placebo actions. *Note: The hypotheses confirmed in the total sample are marked with asterisks. Flags represent the countries in which a given hypothesis was confirmed.*


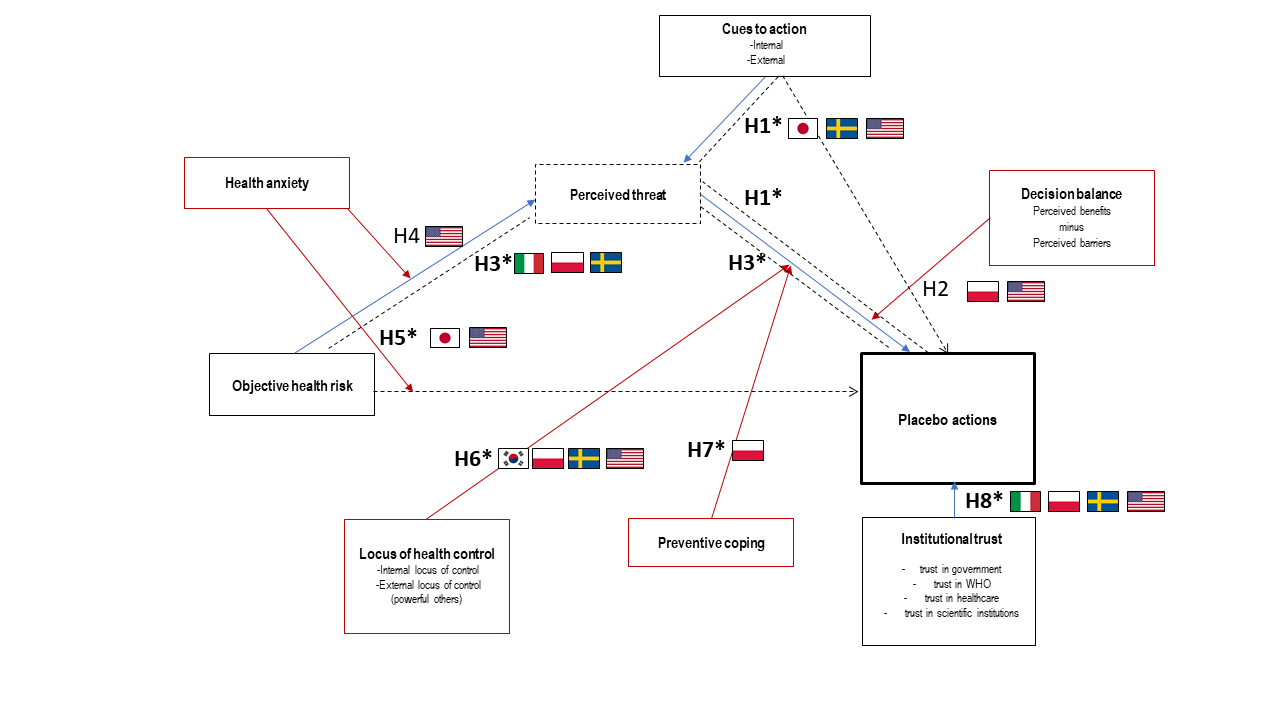


**Supplement I.** Discussion of findings obtained in different nations.

In Japan, neither health anxiety, preventive coping nor locus of control moderated the relationships between subjective risk and the frequency of WHO-recommended or placebo actions. Only the interaction effect of objective risk and health anxiety on the frequency of placebo interventions was significant in this sample. Objective risk itself could have had little impact on the preventive behaviours of Japanese people for a couple of reasons. Although Japan was one of the first countries hit by the coronavirus, the pandemic remained relatively well-contained in terms of the number of cases and mortality rates. At the time of our data collection (May 2020), the infection rate for the whole population was only about 0.01%, and the mortality rate was lower than 0.00001% ^40^. Moreover, the Japanese sample mostly consisted of university students, who are at lower risk of developing serious symptoms of COVID in comparison to older people. Health anxiety has been suggested to lead people to misinterpret information about health risks and to sometimes engage in extreme behaviours to protect their health, especially in the context of viral outbreaks^41^. As stated earlier, the role of this trait may be especially visible in the case of placebo actions which are more freely chosen by individuals and are less influenced by laws and regulations implemented in a given country.

In Italy, the country most severely hit by the COVID-19 pandemic during the first wave (last week of February – first Week of May 2020), which mostly coincided with the collection of data, the engagement in authorities’ recommendations seemed to be driven by the subjective threat posed by COVID-19, especially among individuals who perceived more benefits than barriers associated with preventive actions^4^. High perceived risk also acted as a mediator in the relationship between objective risk and both WHO-recommended and placebo actions. This result may suggest that when people have strong beliefs about the threat posed by coronavirus that are based on both psychological and biological factors, they tend to engage in as many preventive behaviours as possible, following not only WHO recommendations but also implementing placebo interventions^17^.

In Korea, WHO-recommended actions were increased by cues to actions through perceived threat, whereas placebo actions were taken more frequently by individuals with higher internal locus of control. In interpreting this result, the COVID-19 situation in Korea needs to be considered. June 2020, when the data was collected, was during the first wave of the outbreak, and the fear of the disease remained despite the flattening curve at that time. In particular, extensive contact tracking and reporting of the intimate details of COVID-19 cases by Korean authorities made Koreans fearful of social stigma and thus more cautious about their health status. Considering that Koreans are sensitive to saving face^42^, it is understandable that they took extra preventive actions to avoid social stigma, especially when they believed that they had control over the situation (higher internal locus of control). In addition, Koreans’ collectivistic culture may make them hypersensitive to the risk of infecting others^43^ and more willing to try different methods of protection against the disease.

In Poland, higher institutional trust was associated not only with more frequent implementation of WHO recommendations but also with more frequent use of placebo interventions. Previous studies have shown that in this country placebos (like vitamins or homeopathic remedies) are frequently prescribed by primary care physicians, who tend to believe they are effective in reducing symptoms of disease^44,45^. Therefore, it is not surprising that individuals who trust their doctors may use placebo interventions to protect themselves from coronavirus infection. Interestingly, in the Polish subsample the association between subjective threat and placebo usage was stronger when the perceived barriers outweighed the perceived benefits of preventive actions. It seems that when Poles feel endangered by coronavirus and perceive many obstacles to following recommendations, they choose placebo actions that are not only commonly available but also very easy to implement.

The findings in the Swedish sample were mostly consistent with those obtained in the overall sample. Interestingly, Swedes declared the lowest subjective threat in the study, even though this was one of the worst-affected countries, with high excess mortality in older ages when the data collection was initiated (May 2020)^46^. In addition, the Swedish sample had the highest mean age. Considering that age and underlying health problems are the most important risk factors of serious illness and death from COVID-19, this is particularly noteworthy. This finding may be explained by the fact that institutional trust in Sweden is traditionally high^47^, as was also shown in this study. In fact, Sweden showed the highest institutional trust for all institutions (government, WHO, universities) of all the countries included in this study.

The results in the USA sample shared many commonalities with the total sample and supported a variety of the model hypotheses. These findings are not surprising as both theoretical models that inspired the study were developed in Western countries. That said, the USA sample also diverged from the total sample in several interesting ways. For example, subjective threat mediated the relationship between cues to action and placebo actions, a result that was found only in the USA and Japan. The link from objective risk to preventative action, however, was not mediated by subjective threat in the USA. This finding may relate to the strong impact of news and social media on judgments and perceptions about COVID-19 in the USA. Specifically, in the USA the media has provided frequent cues to action as well as inaccurate messages regarding appropriate preventative behaviours^48^. These media cues, rather than objective risk, may impact subjective threat and thereby prevention behaviour. It is also interesting to note that the model’s predictions received greater support for placebo actions than for WHO actions in the USA. Although speculative, this outcome may also be a result of the high transmission of misinformation in the USA regarding effective preventative action, which has possibly increased the use of placebo interventions.

**Supplement J**. Scales created for the purpose of the study

**Subjective threat posed by the illness**

Indicate how much are you afraid that:

1 =very unafraid, 2 = unafraid, 3 = hard to say, 4 = afraid, 5 = very afraid

1. You might be an asymptomatic carrier of the coronavirus
2. You are likely to become infected with the coronavirus
3. You might die as a result of becoming infected with the coronavirus
4. Coronavirus infection could cause irreversible damage to your health
5. In the case of coronavirus infection, you might not get help in time
6. In the case of coronavirus infection, you might be isolated from your close ones
7. People you contact every day could be carriers of coronavirus and pose a threat to your health or life
8. People you pass every day on the street or in shops could be carriers of coronavirus and pose a threat to your health or life
9. Touching objects touched by other people (e.g., door handles, elevator buttons, money) poses a threat to your health or life
10. Being out of home poses a threat to your health or life
11. Touching objects brought from the outside (e.g., groceries, letters, parcels) is a threat to your life and health
12. People you love could get seriously sick or die from the coronavirus.
13. In the case of coronavirus infection, you will lose your job.
14. In the case of coronavirus infection, you may not have enough money to pay your bills or take care of your family.
15. Coronavirus infection might negatively impact your emotions/mental health
16. You might be more susceptible to contracting coronavirus than other people of the same age, gender, and similar health

**Internal and external cues that motivate an individual to take action**

Indicate how true each of the following statements are for you
(from 1 = strongly disagree to 5 = strongly agree):

1. I recognize some COVID-19 symptoms in myself.
2. I know somebody that has been diagnosed with COVID-19 or has been showing symptoms of COVID-19.
3. I keep track of whether people in close proximity to me have been diagnosed with COVID-19.
4. I have been in close proximity to someone who has had coronavirus-like symptoms.
5. People from my environment (family, neighbours, other citizens) follow official recommendations and take preventive actions against coronavirus infection (e.g., wearing masks, washing hands regularly, avoiding unnecessary social contact).
6. People around me are pressuring me to take preventive actions.
7. My family and friends encourage me to take preventive actions.
8. I am obliged to take preventive actions by legal regulations (issued by the government or implemented in my workplace, shops, etc.).
9. I am encouraged to take preventive actions by my government and/or local authorities.
10. I often come across information about COVID-19 in the media.
11. I spend a huge percentage of my time trying to find updates online or on TV about COVID-19.

**Institutional trust:**

Indicate how true each of the following statements are for you (from 1 = strongly disagree to 5 = strongly agree).

Trust in healthcare institutions:

1. Healthcare institutions cover up their mistakes.
2. Healthcare institutions put patients’ needs above keeping medical costs down. (R)
3. Healthcare institutions experiment on patients without them knowing.
4. Healthcare institutions provide good medical care. (R)
5. Healthcare institutions are handling the epidemic well. (R)

Trust in government:

1. My government covers up its mistakes.
2. My government is open and honest with citizens. (R)
3. My government usually has its citizens’ best interests in mind. (R)
4. Most of the time, government decisions are based on competent experts’ recommendations. (R)
5. My government is handling the epidemic well. (R)

Trust in scientific institutions (e.g., universities, research institutes):

1. Scientific institutions (e.g., universities, research institutes) can find solutions to major problems. (R)
2. Scientific institutions (e.g., universities, research institutes) will sacrifice the well-being of others to advance their research.
3. Scientific institutions (e.g., universities, research institutes) care more about their own agenda or reputation than about the truth.
4. Scientific institutions (e.g., universities, research institutes) change their minds about scientific ideas all the time.
5. Scientific institutions (e.g., universities, research institutes) are always open and honest about their work. (R)
6. Scientific institutions (e.g., universities, research institutes) are handling the epidemic well. (R)

Trust in the WHO (World Health Organization)

1. The WHO provides reliable health information. (R)
2. The WHO recommendations regarding disease prevention are trustworthy. (R)
3. The WHO represents the interests of the pharmaceutical industry.
4. The WHO’s decisions are influenced by rich donor countries.
5. The WHO hides important information from the public.
6. The WHO is handling the epidemic well. (R)

**Preventive behaviours undertaken to prevent viral infection**

The following list contains actions that people have used in an effort to prevent COVID-19 infection. We would like to know how often you have used different preventive actions in the last two weeks to protect yourself and others. Please indicate the actions that you have been using specifically in order to prevent COVID-19 infection (not for other purposes).

1 = never, 2 = rarely, 3 = sometimes, 4 = often, 5 = very often

1. Cleaning hands with alcohol-based hand rub. *(WHO)*
2. Taking homeopathic remedies. *(Placebo)*
3. Maintaining at least 1-meter distance from coughing or sneezing people. *(WHO)*
4. Exercising regularly. *(Other)*
5. Taking hot baths. *(Placebo)*
6. Covering mouth and nose with your bent elbow or tissue when coughing or sneezing. *(WHO)*
7. Getting adequate sleep. *(Other)*
8. Practicing physical distancing by avoiding unnecessary travel and staying away from large groups of people. *(WHO)*
9. Using physical methods (e.g., cupping therapy, osteopathy), including stimulating methods (e.g., massage, acupuncture, acupressure). *(Placebo)*
10. Washing hands with soap and water*. (WHO)*
11. Using plants or plant preserves (e.g., garlic, onion, ginger, honey, raspberry juice). *(Placebo)*
12. Avoiding physical contact when greeting. *(WHO)*
13. Wiping surfaces (e.g., desks, tables) and objects (e.g., telephones, keyboards) with a disinfectant. *(WHO)*
14. Avoiding alcohol. *(Other)*
15. Washing fresh products (fruits, vegetables) after grocery shopping. *(WHO)*
16. Wearing face masks in public places*. (WHO)*
17. Avoiding sharing personal items (e.g., telephone, comb) with others. *(Other)*
18. Staying home whenever you can. *(Other)*
19. Choosing to shop for groceries online. *(Other)*
20. Paying by card instead of cash. *(Other)*
21. Using psychological methods (e.g., relaxation, meditation, mindfulness). *(Placebo)*
22. Thorough cooking of meat and eggs. *(WHO)*
23. Staying home if feeling unwell.*(WHO)*
24. Eating a diet high in fruits and vegetables. *(Other)*
25. Refraining from smoking and other activities that weaken the lungs. *(Other)*
26. Trying to minimize stress. *(Other)*
27. Taking dietary supplements (e.g., vitamins, zinc, probiotics). *(Placebo)*
28. Wearing rubber or plastic gloves in public places. *(Other)*
29. Avoiding touching the face (eyes, nose, and mouth*).(WHO)*
30. Using herbal preparations (e.g., teas, tablets, syrups). *(Placebo)*
31. Using essential oils. (*Placebo)*
32. Drinking alcohol. *(Placebo)*
33. Using medications (e.g., antibiotics, aspirin, paracetamol). *(Placebo)*

Do you use other actions (not listed above)?

[If ‘yes’]: Please write down the actions (not listed above) that you have been using and mark on the scale how often you have been using them (enter only one behaviour for each text box).

**Perceived benefits from behaviours**

Indicate how true each of the following statements are for you (from 1 = strongly disagree to 5 = strongly agree):

*Engaging in preventive actions:*

1. keeps me from catching COVID-19.
2. keeps other people around me from catching COVID-19.
3. helps me remain calm.
4. makes people feel safe around me.
5. allows me to do my job despite current circumstances.
6. helps me avoid a penalty for violating the rules.

**Perceived barriers to behaviours:**

*Engaging in preventive actions:*

1. is very time-consuming.
2. costs too much.
3. is uncomfortable / causes me discomfort.
4. is dangerous for my health.
5. makes me a laughing stock.
6. requires too much effort.
7. requires resources that are difficult to come by.
8. requires continuous self-discipline.
9. makes it difficult to do my job.

**Bibliography for supplementary materials**

1. Becker, M. H. & Maiman, L. A. Sociobehavioral Determinants of Compliance with Health and Medical Care Recommendations. *Medical Care* **13**, 10–24 (1975).

2. Rosenstock, I. M. Historical Origins of the Health Belief Model. *Health Education Monographs* **2**, 328–335 (1974).

3. Rosenstock, I. M., Strecher, V. J. & Becker, M. H. Social Learning Theory and the Health Belief Model. *Health Education Quarterly* **15**, 175–183 (1988).

4. Champion, V. L. & Skinner, C. S. The health belief model. in *Health behavior and health education: Theory, research, and practice, 4th ed* 45–65 (Jossey-Bass, 2008).

5. Costa, M. F. Health belief model for coronavirus infection risk determinants. *Rev. Saúde Pública* **54**, (2020).

6. Kim, S. & Kim, S. Analysis of the Impact of Health Beliefs and Resource Factors on Preventive Behaviors against the COVID-19 Pandemic. *International Journal of Environmental Research and Public Health* **17**, 8666 (2020).

7. Mirzaei, A. *et al.* Application of health belief model to predict COVID-19-preventive behaviors among a sample of Iranian adult population. *J Educ Health Promot* **10**, 69 (2021).

8. Tong, K. K., Chen, J. H., Yu, E. W. & Wu, A. M. S. Adherence to COVID-19 Precautionary Measures: Applying the Health Belief Model and Generalised Social Beliefs to a Probability Community Sample. *Applied Psychology: Health and Well-Being* **12**, 1205–1223 (2020).

9. Jones, C. L. *et al.* The Health Belief Model as an Explanatory Framework in Communication Research: Exploring Parallel, Serial, and Moderated Mediation. *Health Communication* **30**, 566–576 (2015).

10. Morgan, F. N., McCabe, D. B., Howley, M. J., McCabe, J. & Steward, M. D. The Influence of Different Types of Cues-to-Action on Vaccination Behavior: An Exploratory Study. *Journal of Marketing Theory and Practice* **18**, 191–208 (2010).

11. Ranjit, Y. S., Shin, H., First, J. M. & Houston, J. B. COVID-19 protective model: the role of threat perceptions and informational cues in influencing behavior. *Journal of Risk Research* **24**, 449–465 (2021).

12. Strecher, V. J. & Rosenstock, I. M. The Health Belief Model. in *Cambridge Handbook of Psychology, Health and Medicine (ed. Baum, A., Newman, A., Weinman, J., West, R. & McManus, C.* 113–117 (Cambridge University Press, 1997).

13. Jahanlou, A. S., Lotfizade, M. & Karami, N. A. *A New Behavioral Model (Health Belief Model Combined with Two Fear Models): Design, Evaluation and Path Analysis of the Role of Variables in Maintaining Behavior*. *Diabetes Mellitus - Insights and Perspectives* (IntechOpen, 2013). doi:10.5772/52966.

14. Lazarus, R. S. & Folkman, S. *Stress, Appraisal, and Coping*. (Springer Publishing Company, 1984).

15. Lazarus, R. S. *Emotion and Adaptation*. (Oxford University Press, 1991).

16. Lazarus, R. S. *Stress and emotion: A new synthesis*. xiv, 342 (Springer Publishing Co, 1999).

17. Biggs, A., Brough, P. & Drummond, S. Lazarus and Folkman’s psychological stress and coping theory. in *The handbook of stress and health: A guide to research and practice* 351–364 (Wiley Blackwell, 2017). doi:10.1002/9781118993811.ch21.

18. Taylor, S. Understanding and treating health anxiety: A cognitive-behavioral approach. *Cognitive and Behavioral Practice* **11**, 112–123 (2004).

19. Kocjan, J. Evaluation of psychometric properties and factor structure of the Polish version of Short Health Anxiety Inventory (SHAI). *Arch Psych Psych* **18**, 68–78 (2016).

20. Cannito, L. *et al.* Health anxiety and attentional bias toward virus-related stimuli during the COVID-19 pandemic. *Sci Rep* **10**, 16476 (2020).

21. Millar, E. B. *et al.* Health anxiety, coping mechanisms and COVID 19: An Indian community sample at week 1 of lockdown. *PLOS ONE* **16**, e0250336 (2021).

22. Dewe, P. & Cooper, G. L. Coping research and measurement in the context of work related stress. in *International Review of Industrial and Organizational Psychology 2007, Vol. 22* 141–191 (John Wiley & Sons Ltd, 2007). doi:10.1002/9780470753378.ch4.

23. Wallston, K. A., Strudler Wallston, B. & DeVellis, R. Development of the Multidimensional Health Locus of Control (MHLC) Scales. *Health Education Monographs* **6**, 160–170 (1978).

24. Cheng, C., Cheung, M. W.-L. & Lo, B. C. Y. Relationship of health locus of control with specific health behaviours and global health appraisal: a meta-analysis and effects of moderators. *Health Psychology Review* **10**, 460–477 (2016).

25. Greenglass, E., Schwarzer, R., Jakubiec, D., Fiksenbaum, L. & Taubert, S. The Proactive Coping Inventory (PCI): A Multidimensional Research Instrument. (1999).

26. Schwarzer, R. & Jerusalem, M. Generalized Self-Efficacy scale. in *Measures in health psychology: A user’s portfolio. Causal and control beliefs.* 35–37 (NFER-NELSON).

27. Almutairi, A. F., BaniMustafa, A., Alessa, Y. M., Almutairi, S. B. & Almaleh, Y. Public Trust and Compliance with the Precautionary Measures Against COVID-19 Employed by Authorities in Saudi Arabia. *Risk Manag Healthc Policy* **13**, 753–760 (2020).

28. Ayalon, L. Trust and Compliance with COVID-19 Preventive Behaviors during the Pandemic. *International Journal of Environmental Research and Public Health* **18**, 2643 (2021).

29. Bargain, O. & Aminjonov, U. Trust and compliance to public health policies in times of COVID-19. *Journal of Public Economics* **192**, 104316 (2020).

30. Caplanova, A., Sivak, R. & Szakadatova, E. Institutional Trust and Compliance with Measures to Fight COVID-19. *Int Adv Econ Res* **27**, 47–60 (2021).

31. Goldstein, D. A. N. & Wiedemann, J. *Who Do You Trust? The Consequences of Political and Social Trust for Public Responsiveness to COVID-19 Orders*. https://papers.ssrn.com/abstract=3580547 (2020) doi:10.2139/ssrn.3580547.

32. Gratz, K. L. *et al.* Adherence to Social Distancing Guidelines Throughout the COVID-19 Pandemic: The Roles of Pseudoscientific Beliefs, Trust, Political Party Affiliation, and Risk Perceptions. *Annals of Behavioral Medicine* **55**, 399–412 (2021).

33. Lalot, F., Heering, M. S., Rullo, M., Travaglino, G. A. & Abrams, D. The dangers of distrustful complacency: Low concern and low political trust combine to undermine compliance with governmental restrictions in the emerging Covid-19 pandemic. *Group Processes & Intergroup Relations* **25**, 106–121 (2022).

34. Nivette, A. *et al.* Non-compliance with COVID-19-related public health measures among young adults in Switzerland: Insights from a longitudinal cohort study. *Social Science & Medicine* **268**, 113370 (2021).

35. Pak, A., McBryde, E. & Adegboye, O. A. Does High Public Trust Amplify Compliance with Stringent COVID-19 Government Health Guidelines? A Multi-country Analysis Using Data from 102,627 Individuals. *Risk Manag Healthc Policy* **14**, 293–302 (2021).

36. Wong, L. *et al.* The role of institutional trust in preventive and treatment-seeking behaviors during the 2019 novel coronavirus (2019-nCoV) outbreak among residents in Hubei, China. (2020) doi:10.1101/2020.02.15.20023333.

37. Ezeibe, C. C. *et al.* Political distrust and the spread of COVID-19 in Nigeria. *Global Public Health* **15**, 1753–1766 (2020).

38. Bavel, J. J. V. *et al.* Using social and behavioural science to support COVID-19 pandemic response. *Nat Hum Behav* **4**, 460–471 (2020).

39. van den Brink-Muinen, A. & Rijken, P. Does trust in health care influence the use of complementary and alternative medicine by chronically ill people? *BMC Public Health* **6**, 188 (2006).

40. Worldometer. COVID-19 CORONAVIRUS PANDEMIC. https://www.worldometers.info/coronavirus/ (2021).

41. Asmundson, G. J. G. & Taylor, S. How health anxiety influences responses to viral outbreaks like COVID-19: What all decision-makers, health authorities, and health care professionals need to know. *J Anxiety Disord* **71**, 102211 (2020).

42. Yang, S. “Chaemyoun-saving (Face saving)” due to Korean Job Loss: Listening to Men’s Voices. *Journal of Comparative Family Studies* **33**, 73–95 (2002).

43. Oh, K.-S. & Lim, S.-W. Subtypes of Social Phobia and Clinical Implication of Offensive Subtype. *Journal of Korean Neuropsychiatric Association* 225–229 (2008).

44. Bąbel, P. The Effect of Question Wording in Questionnaire Surveys on Placebo Use in Clinical Practice. *Eval Health Prof* **35**, 447–461 (2012).

45. Bąbel, P. Use of Placebo Interventions in Primary Care in Poland. *MPP* **22**, 484–488 (2013).

46. Modig, K., Ahlbom, A. & Ebeling, M. Excess mortality from COVID-19: weekly excess death rates by age and sex for Sweden and its most affected region. *European Journal of Public Health* **31**, 17–22 (2021).

47. Holmberg, S. & Rothstein, B. Social trust - The Nordic Gold? *QOG THE QUALITY OF GOVERNMENT INSTITUTE* 1–25 (2020).

48. Geldsetzer, P. Knowledge and Perceptions of COVID-19 Among the General Public in the United States and the United Kingdom: A Cross-sectional Online Survey. *Ann Intern Med* **173**, 157–160 (2020).
